# The Prospective Power of Personality Factors for Family Formation and Dissolution Processes Among Males: Evidence from Swedish Register Data

**DOI:** 10.1007/s10680-025-09748-4

**Published:** 2025-09-16

**Authors:** Steffen Peters

**Affiliations:** 1 Max Planck Institute for Demographic Research, Rostock, Germany; 2 Stockholm University, Stockholm, Sweden

**Keywords:** Family formation, Male fertility, Marital behavior, Personality

## Abstract

Personality has increasingly become relevant for family formation processes. However, the association between personality and family formation (dissolution) has been underexplored in demographic research. This study contributes to existing research by examining the prospective association between two personality factors [social maturity (SM), and emotional stability (ES)] and family formation and dissolution processes, i.e., (1) marital status, (2) fertility, and (3) partnership dissolution as both (a) divorce and (b) cohabitation dissolution, based on large Swedish register data. Poisson regression, Linear Probability, and Cox proportional hazard models were applied for different outcomes. Findings suggest that males with high scores on SM and ES measured at age of assignment to military service (17–20 years) are more likely to get married by age 39 and above. Regarding fertility, SM and ES show positive associations with offspring counts and negative associations with the probability of remaining childless by age 39 and above. Relationship dissolution is negatively linked with SM and ES, in particular among the lowest personality scores. Further analyses using sibling comparisons support these findings.

## Introduction

During the second half of the last century, demographic patterns changed essentially in many European countries. Marriage and fertility rates declined, whereas cohabitation and divorce rates increased (Frejka et al., 2008; Sobotka, 2008; Sobotka & Toulemon, 2008). Several explanations addressing these trends exist, e.g., greater self-realization in fertility due to the availability and use of contraceptives (Frejka, 2008). Personality factors are among those that are most subject to individual differences. Recent research suggests that non-cognitive abilities have become increasingly important for entering parenthood (Aldén et al., 2022), and that personality plays an important role in the marriage market (Dupuy & Galichon, 2014). Therefore, previous studies have increasingly examined the association between personality factors and family-related processes such as marriage (Jokela et al., 2011; Lundberg, 2012), fertility (Allen, 2019; Jokela et al., 2009, 2011; Peters, 2023), or divorce (Boertien & Mortelmans, 2018; Boertien et al., 2017; Lundberg, 2012).

Previous studies examining the relationship between personality and family formation often show essential weaknesses, though. For instance, the analyses of some studies are based on cross-sectional data (Alvergne et al., 2010; Avison & Furnham, 2015) or longitudinal data, but measure personality at relatively late ages (Jokela et al., 2011; Skirbekk & Blekesaune, 2014; Tavares, 2016). This is a strong limitation of previous studies, given that personality may change after crucial life events such as childbirth (Bleidorn et al., 2018; Jokela et al., 2009). One great advantage of the current study is the use of powerful register data from Sweden. Therefore, more detailed analyses on the role of socioeconomic indicators and family background (e.g. via sibling comparisons) for the studied association between personality and family formation are possible.

This study addresses these gaps by examining the prospective association between personality measured in early adulthood, and family formation and dissolution outcomes (marriage, fertility, partnership dissolution) using high-quality Swedish register data. This allows to follow individuals over decades observing their family behavior as well as trajectories in other life domains that may be helpful to understand these associations (e.g., income, education). Furthermore, sibling fixed effects approaches are used to isolate the net influence of personality on family formation and dissolution from background factors (e.g., shared family background) that might bias the relationship.

### Background

Many high-income countries have experienced declines in marriage and fertility rates in the second half of the twentieth century, whereas divorce rates have increased, aligning with the Second Demographic Transition (Lesthaeghe, 2014; van de Kaa, 1987). These trends may be linked with increasing autonomy of the individuals. For instance, they may better plan their fertility based on the availability of contraceptives and abortion. Therefore, personality factors may influence family formation and dissolution processes.

Personality is a broad concept covering ways of thinking, feeling, and behaving (Uher, 2017). It is shaped by both nature (genes) and nurture (social environment) (Kandler, 2012; Kandler & Bleidorn, 2015; Vukasović & Bratko, 2015). Personality factors may determine life course pathways including various life domains (Mencarini et al., 2022) such as educational attainment (Leikas & Salmela-Aro, 2015; Meyer et al., 2019; Usslepp et al., 2020), or labor market trajectories (Damian et al., 2015; Jonason et al., 2018). For instance, the ability to cope with stress or nervousness is beneficial regarding performance in school exams (Chamorro‐Premuzic & Furnham, 2003), or job interviews (Caldwell & Burger, 1998), which may affect later career paths (and thus marriage, fertility, and union dissolution).

Previous studies on the correlation between personality and family formation have primarily used the widely accepted Five Factor Model, often called as Big Five, as personality measure. The five personality factors from this model are agreeableness, conscientiousness, extraversion, neuroticism, and openness. The current study considers two personality factors that are related to the Big Five—social maturity (SM) as *social domain of personality* and emotional stability (ES) as *emotional domain*. SM contains the factors ‘extraversion’ (from the Big Five), responsibility [as a dimension of ‘conscientiousness’ (Roberts et al., 2005; Sutin et al., 2018)], and independence (Bihagen et al., 2013).

ES is the inverse of the factor ‘neuroticism’ (Ashton & Lee, 2005; Goldberg, 1993; McCrae & Costa, 1987) and refers to the capability of dealing with nervousness, stress, and anxiety (Bihagen et al., 2013). Although previous research has used neuroticism, only ES will be addressed here when citing other authors, for simplification purposes.

### Personality and Marriage

Personality may influence the transition to marriage. Previous research has shown that personality is linked with partnership formation (Leone & Hawkins, 2019; Senia et al., 2016), which may result in marriage, eventually. Inequalities in union formation may be based on partner preferences according to personality. For instance, previous studies have shown that socially desired personality factors are also traits that individuals desire in their (potential) partners (Buss & Barnes, 1986; Li et al., 2002). Similarly, indicators of the social dimension of personality are associated with higher levels of closeness to, and importance of, friends (Neyer & Asendorpf, 2001), and with an increasing interest in short-term mating (Schmitt & Shackelford, 2008). Moreover, social capacities are positively linked with the chances of falling in love at a younger age (Asendorpf & Wilpers, 1998). Once being in a romantic relationship, people with higher social competences report higher partnership satisfaction (Orth, 2013) and quality (Holland & Roisman, 2008), which may increase the probability of getting married.

Regarding empirical evidence, previous studies have suggested positive associations between personality traits that are related to social competences and the probability to get married, particularly among males (Jokela et al., 2011; Lundberg, 2012). Furthermore, these traits are positively linked with an earlier entry into marriage (Jokela et al., 2011). Therefore, the first hypothesis assumes a positive relationship between social maturity and the probability of having ever been married.

The emotional dimension of personality may also be linked with the entry into marriage. Young individuals with higher emotional stability (ES) are in demand in the partner market (Botwin et al., 1997; Dupuy & Galichon, 2014; Figueredo et al., 2006), which may increase the chances of getting married. Furthermore, previous research has suggested a positive association between ES and both partnership satisfaction (Karney & Bradbury, 1997; Malouff et al., 2010; McNulty, 2008) and relationship quality (Donnellan et al., 2004). Findings from previous research on the link between ES and marital behavior are mixed, though. For instance, no association between ES and marriage probability has been found in Germany (Lundberg, 2012), but a positive one among men from the U.S. (Jokela et al., 2011). Consequently, the second hypothesis claims that ES and the probability of having ever been married are positively associated.

### Personality and Fertility

Personality may also predict fertility outcomes, e.g., via intentions or expectations, which are (directly) linked with fertility (Ajzen & Klobas, 2013; Miller, 2011). Indeed, indicators of social personality traits are associated with parenthood expectations (Hutteman et al., 2013) and fertility intentions (Miller, 1992). Furthermore, children may increase the number of social interactions since additional persons are living in the household. This may motivate social individuals to have children. Contrary, opposite effects of parenthood may also emerge when one’s social life, including activities with friends, is restricted since one may need to take care of offspring instead. Furthermore, social personality factors may be linked with fertility through sexual behavior. For example, higher social competences are positively associated with the number of sexual partners (Allen & Desille, 2017; Miller et al., 2004; Nettle, 2005, 2006; Schmitt, 2004).

Previous research strongly suggests a positive association between social personality indicators and childbearing. Indicators of social competences are positively related to a higher likelihood of having a first and second child (Jokela et al., 2009, 2011), accelerated childbearing (Jokela et al., 2011; Tavares, 2016), and lower risk of remaining childless (Avison & Furnham, 2015) among both men and women. However, several findings indicate stronger associations among males (Allen, 2019; Jokela et al., 2011; Peters, 2023; Skirbekk & Blekesaune, 2014). Based on the elaborations above, a positive association between SM and fertility is expected.

The emotional dimension of personality may also shape fertility outcomes since ES is related to decreasing ambivalence regarding fertility decisions (Pinquart et al., 2008), and to lower depression risks (Gershuny & Sher, 1998). Entry into parenthood increases stress levels (Epifanio et al., 2015), and negatively affects social life (Johnson & Rodgers, 2006) and psychological well-being (McLanahan & Adams, 1987), which may refrain people with low ES from entering parenthood. On the other hand, individuals with low ES scores may consider parenthood as a stabilizing factor in a partnership (Friedman et al., 1994), and in life more generally (Johns et al., 2011).

Previous research provides evidence for positive associations between ES and childbearing. Positive links can be detected between ES and becoming a parent (Jokela, 2012), or having a second and a third child (Jokela et al., 2009). Some evidence indicates that ES plays a greater role for women’s offspring counts and remains relatively unimportant among men (Jokela et al., 2011). Recent research, however, shows that the positive effects of ES on completed fertility have particularly risen among men across decades in the Nordic context (Skirbekk et al., 2025). Therefore, a positive association between ES and childbearing is expected in this study.

### Personality and Union Dissolution

Apart from family formation processes, personality may also be associated with union dissolution. Social personality traits may also attract opposite-sex persons outside the partnership, which may lead to higher infidelity (Orzeck & Lung, 2005), potentially resulting in higher dissolution risks. Contrary, they are positively linked with partnership quality and satisfaction (see above), indicating greater relationship stability and lower dissolution risks.

Consequently, research on social competences and divorce risks shows mixed evidence, including positive (Boertien & Mortelmans, 2018; Boertien et al., 2017; Lundberg, 2012), or no associations (Le et al., 2010; Zare et al., 2013). Thus, no hypothesis on the association between SM and dissolution can be set up.

Dissolution risks may particularly be based on emotional conditions of the partners. Individuals with a poor emotional constitution may worry a lot about many life facets, including their own partnership, which may result in lower partnership satisfaction (Fisher & McNulty, 2008; Spikic & Mortelmans, 2021). Furthermore, lower ES of a partner is linked with higher infidelity risks (Orzeck & Lung, 2005), which may also contribute to higher dissolution risks. On the other hand, low ES is also linked with higher attachment anxiety (Noftle & Shaver, 2006), that is, such individuals may worry about connecting with new networks after a potential partnership dissolution (Spikic & Mortelmans, 2021). This may motivate people with low ES to stay even in an unhappy partnership.

If personality effects on divorce have been found, the strongest associations have referred to emotional stability (Spikic & Mortelmans, 2021). Low scores on ES are linked with higher divorce risks (Boertien & Mortelmans, 2018; Lundberg, 2012; Roberts et al., 2007), and higher risks of relationship dissolution (Solomon & Jackson, 2014). Additionally, personality traits related to low ES show higher divorce risks (Jockin et al., 1996). Therefore, a negative association between ES and dissolution risks is expected for the present analyses, although some evidence suggests that ES is not linked with divorce risks (Boertien et al., 2017).

### The Role of Socioeconomic Status and Family Background

Previous studies have suggested that socioeconomic status (SES) may play an important role for the link between personality and family formation. Social and emotional dimensions of personality have been linked with job interview performance (Caldwell & Burger, 1998; Cook et al., 2000), educational attainment (Damian et al., 2015), annual income (Jonason et al., 2018) and occupational attainment (Roberts et al., 2007). However, social competences have also been linked with a lower likelihood to follow the educational path after upper secondary education (Usslepp et al., 2020), indicating lower SES attainment, eventually. Furthermore, ES is positively associated with the tendency to define clear goals and work efficiently (Judge & Ilies, 2002), and with career success (Judge et al., 1999).

SES indicators are associated with marital behavior in the Nordic countries (Duvander & Kridahl, 2020; Sandström & Stanfors, 2020). For instance, high education is positively linked with the intention to marry among males (Wiik et al., 2010). The correlation between income and marital behavior seems to be less clear, though. On the one hand, there is evidence from Sweden indicating that income does not play a great role in marriage intentions (Duvander & Kridahl, 2020). However, another study suggests that income is positively linked with marriage intentions among males from Nordic countries (Wiik et al., 2010). A negative association between SES and relationship dissolution has been found using data from Norway (Lyngstad, 2004) and Finland (Jalovaara, 2001, 2013), although the consideration of a partner’s characteristics may attenuate this pattern (Jalovaara, 2003).

SES may also shape fertility behavior since a higher SES may be more attractive to a potential partner due to greater access to resources and the provision of protection (Buss, 1994, 2006; Cummins, 2006). Previous research has found a positive association between SES indicators and fertility among males (Fieder et al., 2005; Hopcroft, 2006), in particular in the Nordic countries (Kolk, 2019; Kolk & Barclay, 2021). For instance, income is positively linked with second and third childbirths among males in Sweden (Andersson & Scott, 2007). Additionally, lower-educated men from Nordic countries show lower fertility and higher probabilities to remain childless over their life course (Jalovaara et al., 2019). Considering previous findings on SES and marriage/fertility, it is crucial to include factors such as income and education in the analyses of this study.

Furthermore, family background information is associated with both personality (e.g., Jokela et al., 2017) and family formation (e.g. Kramarz et al., 2021) and may, therefore, confound the relationship between personality and family formation processes. For instance, sociability is positively linked with maternal education, and negatively associated with sibling group size (Jokela et al., 2017). Some research also suggests that genetics may influence personality traits (Penke & Jokela, 2016; Penke et al., 2007; Van Gestel & Van Broeckhoven, 2003). Additionally, genetics shape the association between personality and marriage (Johnson et al., 2004).

Numerous studies have further found that individuals show similar fertility patterns as their siblings (Buyukkececi & Leopold, 2021; Dahlberg & Kolk, 2018; Lyngstad & Prskawetz, 2010). Cools and Hart (2017) have found that males’ fertility increases by each additional sibling. The positive association between number of siblings and own fertility appears to be stronger among firstborns compared to later born siblings (Morosow & Kolk, 2020). Kolk (2015), however, has found that the number of siblings does not have a causal effect on completed fertility in Sweden. These findings suggest that family background (parental education, sibling group size, siblings’ family formation processes) may influence one’s own family formation, and personality effects may be partly explained by considering shared background information.

Additionally, family background is linked with marital dissolution (Bumpass et al., 1991; Dronkers & Hox, 2006). For instance, genetics have been found to shape divorce risks (McGue & Lykken, 1992). Furthermore, parental factors such as social class or maternal employment status have been associated with divorce risks (Todesco, 2013). It is, therefore, crucial to adjust for family background factors, which is done through the application of sibling fixed effects models and adjustment for observables in this study. Moreover, specific barriers exist, which may result in decreasing dissolution risks, such as the presence of children (Knoester & Booth, 2000; Todesco, 2011). Consequently, patterns in the association between personality and divorce risks will additionally be examined by parental status.

## Data and Methods

The analyses of this study are based on Swedish register data. Each resident in Sweden has a unique identification number, through which information from various registers can be linked. Marital behavior, fertility, and educational level have been collected in civil registers, income in tax registers that further define cohabitation in the analyses. Each individual has to report the current address to the tax office, which is the most specific information on area of residence in the Swedish registers (Thomson & Eriksson, 2013). Possibly, individuals live at the same address but not share a household (e.g., student dorms). However, if a man and a woman have a joint child together and are registered at the same property number, one can strongly assume cohabitation (Thomson & Eriksson, 2013).

Personality factors and cognitive skills come from military conscription data and are available between 1983 and 1997. All young men were obliged to take the military tests during this time. Information on family background was drawn from multigenerational registers. Only full siblings with same mothers and fathers were included in the within-family analyses (fixed effects). These models control for unobserved heterogeneity between individuals to the extent that they are shared by siblings (e.g., genetics, parental background).

The analytical sample is restricted to a relatively homogeneous group of men who were between 17 and 20 years of age at time of recruitment. This includes the vast majority of the male population (98%) from the birth cohorts 1963–1979. All men who left Sweden or died by age 39 have been excluded. At the time of last observation (2018), males were between 39 and 55 years of age. This is a reasonable threshold for fertility analyses, as fertility patterns do not change much among Swedish men after age 40 (Barclay & Kolk, 2020; Nisén et al., 2014). The analytical sample on marriage and fertility consists of 651,790 males (sibling comparison: 222,848). Divorce models were run for males who had married by 2018 (390,344 men, sibling comparison: 136,414). Analyses on cohabitation based on joint childbearing (only non-married males) have been run for 129,820 men (sibling comparison: 42,055). All models include either SM or ES in order to avoid multicollinearity, based the Pearson’s correlation coefficient of 0.65, which indicates a moderate or even strong correlation between both personality factors.

### Personality Factors from Psychological Interviews

Social maturity (SM) and emotional stability (ES) stem from 20–30 min semi-structured interviews by licensed psychologists during obligatory military conscription (Ludvigsson et al., 2022). Both factors are available as scores from 1 (“Low”) to 5 (“High”), and have been collected for all men at the time of their recruitment.

SM includes factors such as extraversion, independence, and responsibility (Bihagen et al., 2013; Mood et al., 2012). In recent conscription cohorts, the military psychologists were required to ask the young men to evaluate their relationships to teachers and classmates, and whether they are interested in social activities including sports or outdoor life (Ludvigsson et al., 2022). Although questions might have changed over time and specific question formulations were left with the psychologists, it suggests that the military has been strongly interested in social skills and activities of the males. Social skills are important for serving in the army; in particular in officer positions, to accompany recruits during their military service, or to keep the group’s motivation high (Larsson & Kallenberg, 2006). Officers with high social skills tend to be sociable, talkative, and easy-going, and may, therefore, connect easily with their team members (Larsson & Kallenberg, 2006). Recruits may be more loyal and willing to take risks within a trustful relationship to their team leader (Grönqvist & Lindqvist, 2015), which is desired by the army.

Emotional stability includes stress resilience, nervousness, and anxiety (Bihagen et al., 2013; Mood et al., 2012). In the latest conscription cohorts, the males were asked whether they have problems in controlling their temper or how they manage stress (Ludvigsson et al., 2022), which may also reflect previous interests of the army in the emotional skills of the males. The capacity to maintain composure and control over one’s emotions can be useful in stressful situations within the military (Larsson & Kallenberg, 2006). In particular, officers may benefit from high levels of emotional stability since stressful situations require wise decisions and clear instructions from strong leaders (Grönqvist & Lindqvist, 2015).

### The Swedish Military Conscription and Its Goals

The recruitment procedure has been identical for all males, and consisted of several steps, including medical tests (e.g. blood pressure) and physical tests (e.g. hearing capability) (Mönstringshandboken, 2021). At an early stage of the conscription, the young men had to undergo a cognitive ability test that included tasks on logical, spatial, verbal, and technical abilities (Kolk & Barclay, 2019). Subtest scores were transformed onto a general stanine scale (mean: 5; standard deviation: 2) (Kolk & Barclay, 2019). Toward the end of the conscription, psychological interviews took place (Mönstringshandboken, 2021). Psychologists had prior test results available (Lindqvist & Vestman, 2011; Ludvigsson et al., 2022; Nyberg et al., 2020).

One of the main goals of the army was to filter suitable candidates for different positions in the military, and deselect unsuitable men (e.g., neurotics, psychopaths) (Ludvigsson et al., 2022). Almost one third of the recruits of each cohort was trained as officers in lower hierarchies who stay in the military for approximately one year (Grönqvist & Lindqvist, 2015). The Swedish military desires certain personality factors and skills among its officers (Grönqvist & Lindqvist, 2015), potentially to increase team performances, similar to evidence from previous research (Chidester et al., 1991). For instance, only recruits with IQ scores of 5 or higher are considered for different officer positions (Grönqvist & Lindqvist, 2015).

### Reliability of Ratings by Psychologists

The Swedish military has addressed the question of interrater reliability in several ways. First, only certified psychologists with an educational degree have been chosen (Lindqvist & Vestman, 2010). Second, they were trained in a 4-weeks program at the Swedish National Service Administration for their military tasks (Ludvigsson et al., 2022; Nyberg et al., 2020). Moreover, standardized guidelines were available for the interviews with the recruits (Lindqvist & Vestman, 2010). These guidelines have not been made available to the public (Nilsson et al., 2001; Nyberg et al., 2020). However, previous studies provide some details, at least. For instance, the psychologists should focus on personality factors only, without considering the motivation for attending military service (Lindqvist & Vestman, 2010; Ludvigsson et al., 2022), which may partly prevent interview manipulation by the recruits if they do not intend to serve as military officer (Lindqvist & Vestman, 2010). Furthermore, previous research has examined interrater discrepancies. Psychologists assessed recruits from interviews that were recorded, but conducted by other psychologists, and interrater reliability was relatively high (Nyberg et al., 2020). This is supported by similar personality mean values and standard deviations across counties for the present sample (Table 2 in the Appendix). Further information on the military conscription, the psychological interview, the psychological measures and the advantages over self-reports is shown in the Appendix.

### Outcomes

This study considers several outcomes. First, marital status by age 39 and above (0—“Never married”, 1—“Ever married”) is examined. Second, completed fertility by age 39 and above as both the number of children (0–22) and childlessness (0—“At least one child;” 1—“Childless”) is of interest. Third, divorce risk over time (event: 0—“Not divorced”, 1—“Divorced”) is examined for all married men by age 39 and above (risk starts at marriage). Finally, analyses on cohabitation dissolution risk over time based on males who live together with the mother of their child (event: 0—“Not separated,” 1—“Separated;” risk starts at entry into fatherhood) are run.

### Control Variables

The analyses of this study control for a set of variables. All models include cognitive skills (1 “Low” to 9 “High”), birth year (1963–1979), birth order, and sibling group size (including sisters). Additionally, the role of SES indicators has been explored. Indicators are highest educational level obtained by age 39 (1 “No Basic Education”, 2 “Primary”, 3 “Lower Secondary”, 4 “Upper Secondary”, 5 “Post-Secondary”, 6 “Tertiary”, 7 “Doctor”) and income (cumulated by age 39).

### Statistical Models

Linear probability models (LPM) are applied in order to examine marital status by age 39 and above. The LPM for the analyses take the following forms:

1 $$Pr\left({Y}_{i}=1|{X}_{i}={x}_{i}\right)={\beta }_{0}+{\beta }_{1}{personality}_{i}+{\beta }_{2}{cognitive}_{i}+{\beta }_{3}{birth\_year}_{i}+{\beta }_{4}{birth\_order}_{i}+{\beta }_{5}{sibling\_group\_size}_{i}$$

2 $$Pr\left({Y}_{i}=1|{X}_{i}={x}_{i}\right)={\beta }_{0}+{\beta }_{1}{personality}_{i}+{\beta }_{2}{cognitive}_{i}+{\beta }_{3}{birth\_year}_{i}+{\beta }_{4}{birth\_order}_{i}+{\beta }_{5}{sibling\_group\_size}_{i}+{\beta }_{6}{\text{ln}(income)}_{i}+{\beta }_{7}{education}_{i}$$

The probability of having ever been married, the outcome of Eqs. (1) and (2), for each individual *i* depends on a set of variables. The intercept *β*_*0*_ describes the baseline value of the model. *Personality* refers to the corresponding personality factor (SM or ES). Cognitive skills are included as the categorical variable *cognitive*. Furthermore, models control for birth cohorts (*birth_year*), birth order among all siblings in the family (*birth_order*), and the total number of siblings, including the individual (*sibling_group_size*). Sibling group size has been excluded as covariate from the fixed effects analyses since this information is shared between brothers. Equation (2) also includes SES factors. *Income* means the standardized logarithm of the cumulated income by age 39. *Education* represents the highest educational level by age 39.

Offspring counts have been analyzed by Poisson regression models. Formally, this approach is described as follows:

3 $$log\left(E\left[{Y}_{i}|{X}_{i}\right]\right)={\beta }_{0}+{\beta }_{1}{personality}_{i}+{\beta }_{2}{cognitive}_{i}+{\beta }_{3}{birth\_year}_{i}+{\beta }_{4}{birth\_order}_{i}+{\beta }_{5}{sibling\_group\_size}_{i}$$

4 $$log\left(E\left[{Y}_{i}|{X}_{i}\right]\right)={\beta }_{0}+{\beta }_{1}{personality}_{i}+{\beta }_{2}{cognitive}_{i}+{\beta }_{3}{birth\_year}_{i}+{\beta }_{4}{birth\_order}_{i}+{\beta }_{5}{sibling\_group\_size}_{i}+{\beta }_{6}{\text{log}(income)}_{i}+{\beta }_{7}{education}_{i}$$

The logarithm of the expected number of children *Y* for each individual *i* depends on the vector of explanatories *X*. Control variables are identical to those in Eqs. (1) and (2). Again, sibling group size was eliminated as a covariate from fixed effects models since these require variation in all covariates by default. Analyses on childlessness are based on LPM as shown in Eqs. (1) and (2), including the identical covariates, just with a different outcome (probability of being childless at age 39 and above).

Risks of partnership dissolution (divorce and cohabitation dissolution among parents) are examined using Cox Proportional Hazard models. The underlying time scales are time since marriage and time since first childbirth within an assumed cohabitation, respectively. The observation ends with time at dissolution or the end of the study (2018)—whichever comes first. The model censors the data since not all individuals have experienced the event of interest by the end of the observation time. The models are shown in Eqs. (5) and (6):

5 $$h\left(t|{x}_{i}\right)= {h}_{0}\left(t\right)exp\left\{{\beta }_{0}+{\beta }_{1}{personality}_{i}+{\beta }_{2}{cognitive}_{i}+{\beta }_{3}{birth\_year}_{i}+{\beta }_{4}{birth\_order}_{i}+{\beta }_{5}{sibling\_group\_size}_{i}\right\}$$

6 $$h\left(t|{x}_{i}\right)= {h}_{0}\left(t\right)exp\left\{{\beta }_{0}+{\beta }_{1}{personality}_{i}+{\beta }_{2}{cognitive}_{i}+{\beta }_{3}{birth\_year}_{i}+{\beta }_{4}{birth\_order}_{i}+{\beta }_{5}{sibling\_group\_size}_{i}+{\beta }_{6}{\text{ln}(income)}_{i,t-1}+{\beta }_{7}{education}_{i,t-1}\right\}$$

The hazard *h* on each time point *t* depends on the vector of independent variables *x* for each individual *i*. It is the product of the baseline hazard *h*_*0*_ (time-variant) and the exponentiated sum of the estimated intercept (*β*_*0*_) and the independent variables *x*, which are multiplied by the corresponding coefficients* β*. The set of explanatories is identical to those in Eqs. (1) and (2) above. However, education and income were included as both time-varying and lagged variables (i.e., information from year t−1). Furthermore, based on parenthood as a potential barrier to divorce, additional analyses on divorce risks, including an interaction between personality and parental status (yes/no, time-varying), were conducted.

## Results

### Descriptives

Among men from birth cohorts 1963–1979, 40.94% have never been married by age 39, and 59.06% have ever been married. About one fifth (20.64%) stayed childless, 14.76% had one child, 42.81% two, and 16.71% three children. Both personality factors follow a relatively normal distribution, with largest numbers on the mid-score 3 (SM: 44.73%; ES: 49.43%). Since the group of missing values is relatively large for both SM (19.58%) and ES (19.65%), a separate category including missing values was kept in the analyses. Most recruits scored 4 (15.35%), 5 (23.64%), or 6 (16.62%) on cognitive skills, and obtained a lower secondary (31.18%), upper secondary (22.85%), or tertiary degree (21.17%). Further information is shown in Table 3 in the Appendix.

Table 1 shows descriptive statistics for the different analytical samples (marriage/fertility, divorce, cohabitation dissolution). The proportion of men who have ever been married by age 39 and higher increases with higher scores of SM (score 1: 0.38; score 5: 0.75). The average overall offspring count in the analytical sample is 1.73. This value varies between 1.37 and 2.03 by SM scores, with higher SM showing higher fertility. Furthermore, males with lower SM scores are more likely to remain childless (e.g., 37% among males with score 1) than males with highest SM scores (e.g., 10% among men with score 5). Higher SM scores also show higher levels of IQ, education, and income. Similar patterns emerge for ES. Furthermore, partnership dissolution risks decrease over SM and ES scores, as shown in the bottom part of Table 1.

**Table 1:**
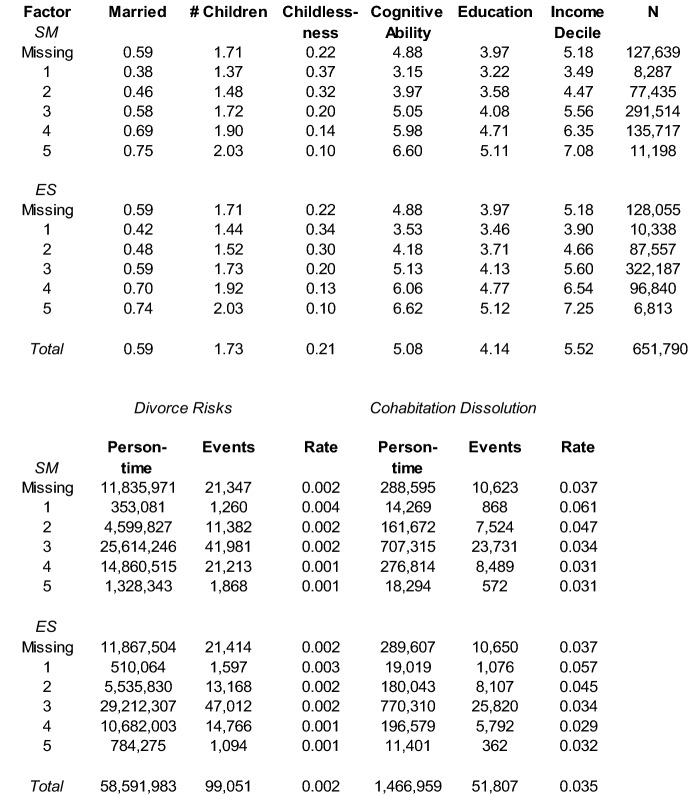
Mean values according to SM and ES

### Findings on Personality and Marriage

**Fig. 1:**
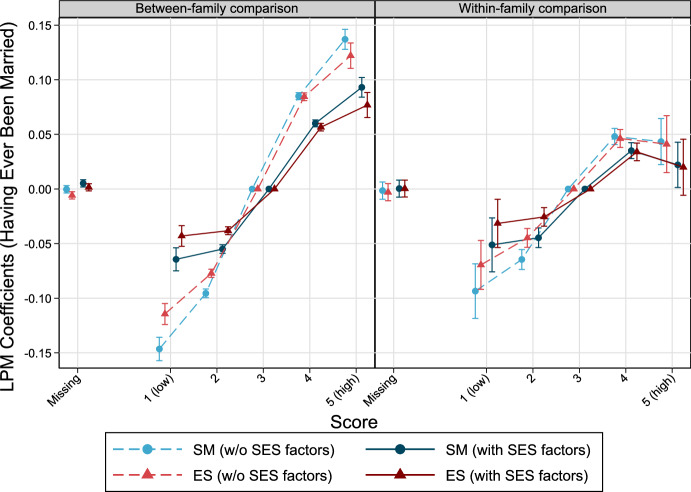
The relationship between personality factors measured at ages 17–20 and the probability to get married by age 39 and higher among Swedish men born 1963–1979. Linear probability models, error bars are 95% confidence intervals. *Note* Models without SES factors control for cognitive abilities, birth year, birth order, and in case of between-family considerations for sibling group size. Models with SES factors include income and education, additionally

SM and ES are positively linked with the probability of having ever been married by age 39 and above (Fig. 1). In models without SES factors, males with the highest SM score (5) show an almost 14% higher probability of having ever been married compared to males with the reference score of 3, holding all other covariates constant. This is an increase of approximately 23.2% compared to the baseline probability (59%). Conversely, men with the lowest SM (1) are 14.6% less likely to get married by age 39 and above, which is a reduction of ca. 24.7% compared to the baseline probability in the entire sample. Estimates regarding ES are very similar, albeit on a slightly lower level. Associations attenuate when income and education are included in the models but general patterns remain. When brothers are compared to each other (within-family comparison) the magnitudes decrease slightly but, again, the general patterns persist, although the marriage probability no longer changes much across the highest personality scores. Regression model estimates are shown in Tables 4 (SM) and 5 (ES). Results do not change much across models with and without IQ, as can be seen in the Appendix (Fig. 6).

### Findings on Personality and Fertility

**Fig. 2:**
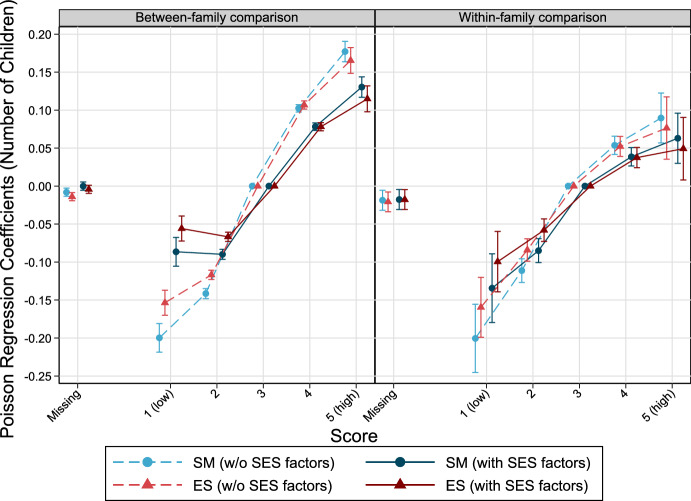
The relationship between personality factors measured at ages 17–20 and number of children by age 39 and higher among Swedish men born 1963–1979. Poisson regression models, error bars are 95% confidence intervals. *Note* Models without SES factors control for cognitive abilities, birth year, birth order, and in case of between-family considerations for sibling group size. Models with SES factors include income and education, additionally

Findings from Poisson regression models indicate that SM and ES are positively associated with the number of children (Fig. 2). Models without SES factors reveal stronger positive associations with fertility for both SM and ES. For instance, men with lowest SM scores obtain 0.20 fewer children on average by age 39 and above compared to the ones who scored 3, conditioned on all other covariates being fixed. However, the magnitude of this coefficient turned to − 0.09 when income and education were included. Compared to the overall average of offspring counts in the total population (1.73), this means a reduction of ca. 5%. Patterns regarding ES are very similar. Comparison between brothers (within-family analyses) do not change the results much. All coefficients from the Poisson regression analyses can be found in the appendix (Tables 6 and 7).

**Fig. 3:**
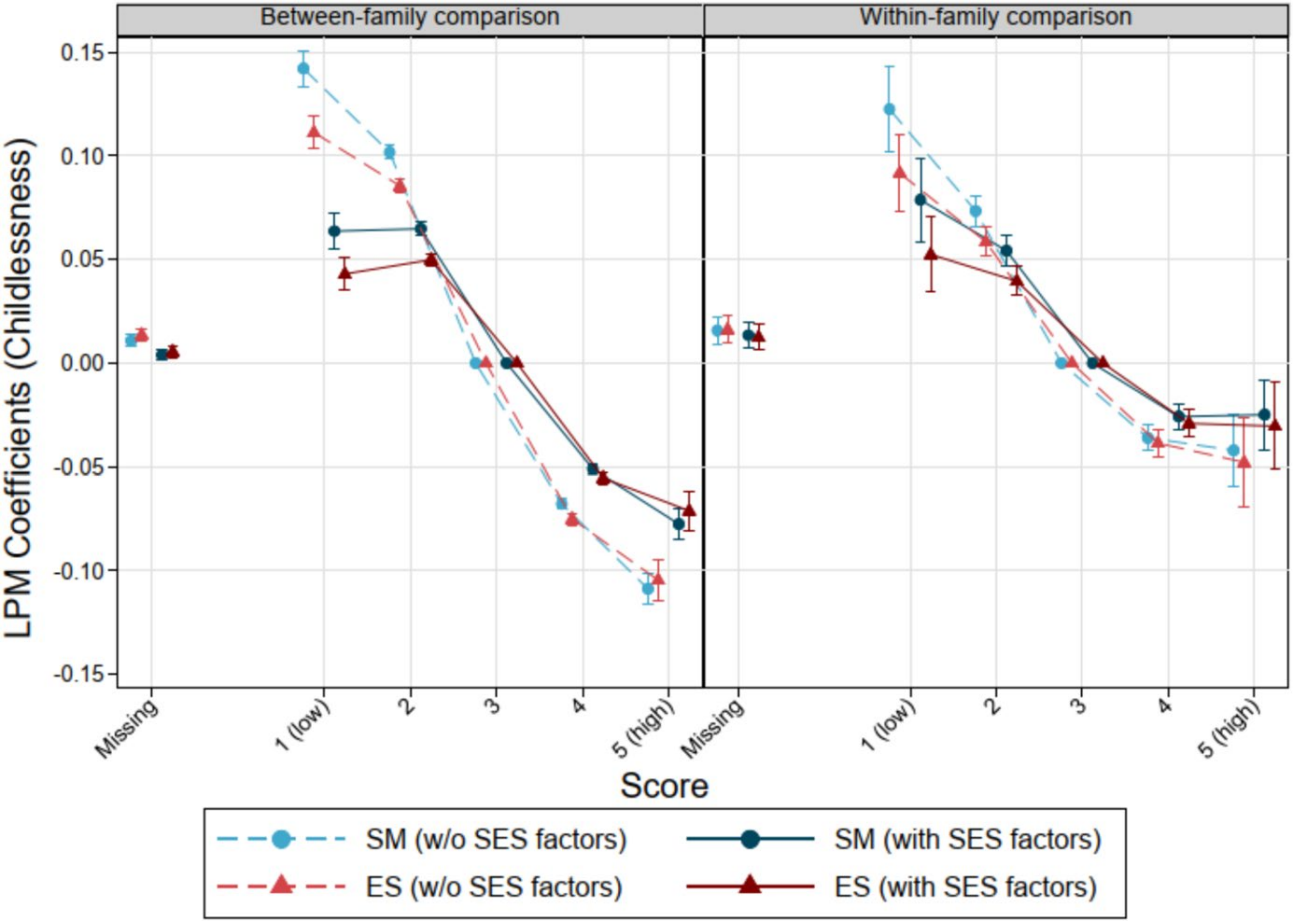
The relationship between personality factors measured at ages 17–20 and childlessness by age 39 and higher among Swedish men born 1963–1979. Linear probability models, error bars are 95% confidence intervals. *Note* Models without SES factors control for cognitive abilities, birth year, birth order, and in case of between-family considerations for sibling group size. Models with SES factors include income and education, additionally

Figure 3 depicts the negative association between both personality factors and childlessness. For instance, a young male with an SM score of 1 shows a more than 6% higher probability of remaining childless by age 39 and above (after including income and education), compared to another young male with an SM score of 3 (given that all other covariates are kept constant). This represents a 30% higher probability than the baseline level (0.21) of remaining childless. In contrast, a male with an SM score of 5 shows a more than 7% lower probability of remaining childless. Results for ES reveal similar patterns. Findings for both personality factors persist in brother comparisons, despite higher statistical uncertainties (Tables 8 and 9). Models with and without IQ reveal similar estimates as can be seen in Figs. 7 and 8 in the Appendix. Additionally, coefficients from models without SES indicators are up to twice as large as the findings from the full models in the between-family comparisons, suggesting an important mediating role of SES for the personality-childlessness link.

### Findings on Personality and Divorce

**Fig. 4:**
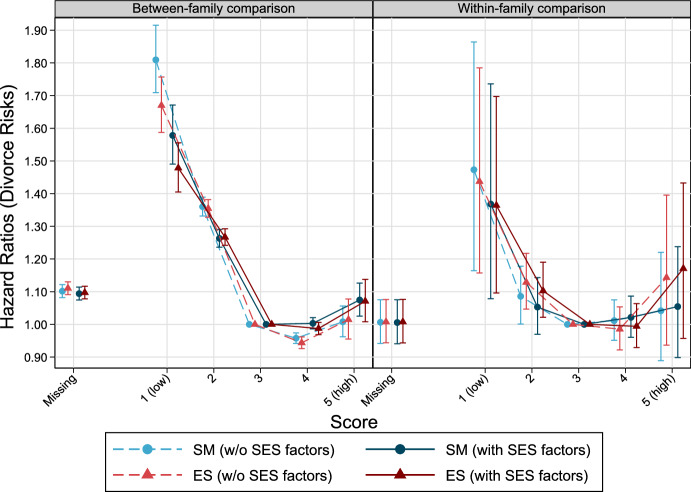
The relationship between personality factors measured at ages 17–20 and divorce risks by age 39 and higher among Swedish men born 1963–1979. Cox Proportional Hazards models, error bars are 95% confidence intervals. *Note* Models without SES factors control for cognitive abilities, birth year, birth order, and in case of between-family considerations for sibling group size. Models with SES factors include income and education, additionally

Figure 4 depicts the association between SM/ES and divorce risks among men who ever married. Considering between-family analyses, married men with low scores (1 and 2) on SM and ES show higher divorce risks compared to the reference group (score 3). For instance, males who received an SM of 1 at the time of military conscription and entered marriage before age 39, show a more than 80% higher risk of getting divorced over time (all other covariates held constant). Including income and education reduces this risk to 58%. Highest scores on both SM and ES show slightly higher divorce risks (7–8%) than the reference group (score 3). Similar patterns (but on a lower level) emerge when brothers are compared to each other (Fig. 4); however, statistical uncertainty increases (Tables 10 and 11). To examine potential differences between parents and non-parents, models including an interaction between personality and parental status (time-varying) were run. No large differences in the overall patterns on the personality-divorce association between parents and non-parents were detected, i.e., decreasing divorce risk among lowest personality scores and no large changes across highest scores were found. In general, however, parents show a lower divorce risk. Among non-parents, increasing divorce risk among higher personality scores might be suggested, but statistical uncertainty is relatively large for this group (Fig. 9).

### Findings on Personality and Cohabitation Dissolution

**Fig. 5:**
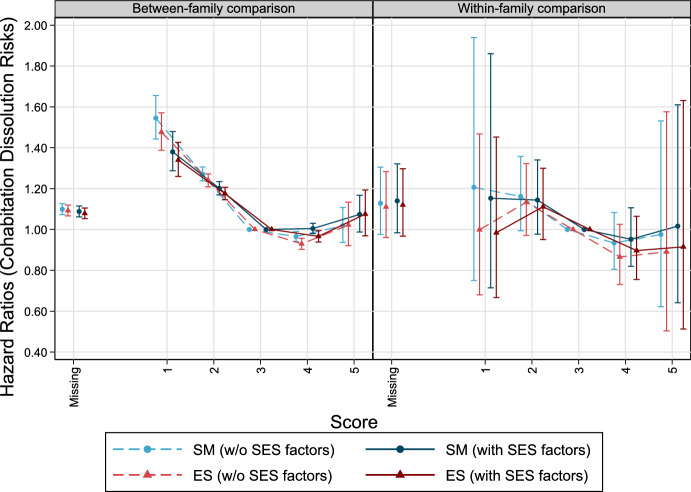
The relationship between personality factors measured at ages 17–20 and cohabitation dissolution risks by age 39 and higher among Swedish men born 1963–1979. Cox Proportional Hazards models, error bars are 95% confidence intervals. *Note* Models without SES factors control for cognitive abilities, birth year, birth order, and in case of between-family considerations for sibling group size. Models with SES factors include income and education, additionally

**Fig. 6:**
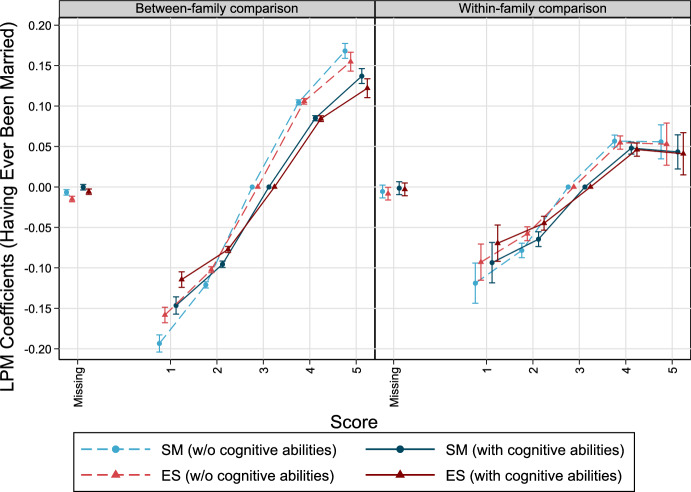
The relationship between social maturity measured at ages 17–20 and the probability to get married by age 39 and higher among Swedish men born 1963–1979. Linear probability models, error bars are 95% confidence intervals. *Note* Models without cognitive abilities control for birth year, birth order, and in case of between-family considerations for sibling group size. Models with cognitive abilities include cognitive abilities, additionally

**Fig. 7:**
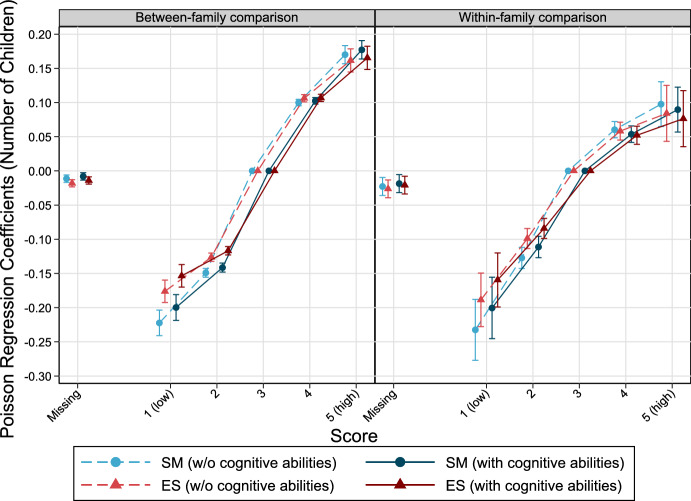
The relationship between personality factors measured at ages 17–20 and number of children by age 39 and higher among Swedish men born 1963–1979. Poisson regression models, error bars are 95% confidence intervals. *Note* Models without cognitive abilities control for birth year, birth order, and in case of between-family considerations for sibling group size. Models with cognitive abilities include cognitive abilities, additionally

**Fig. 8:**
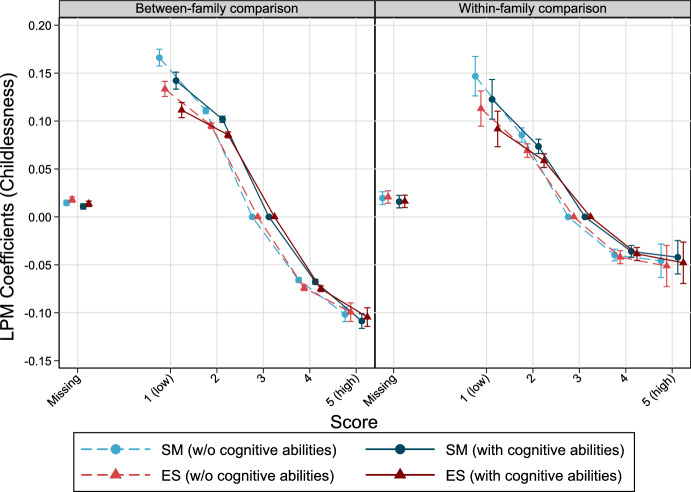
The relationship between personality factors measured at ages 17–20 and childlessness by age 39 and higher among Swedish men born 1963–1979. Linear probability models, error bars are 95% confidence intervals. *Note* Models without cognitive abilities control for birth year, birth order, and in case of between-family considerations for sibling group size. Models with cognitive abilities include cognitive abilities, additionally

Furthermore, the association between both personality factors and cohabitation dissolution for individuals who live with a partner based on joint parenthood (marriages excluded) is examined. Results are shown in Fig. 5. Low SM and ES scores are linked with higher dissolution risks among parents. Including SES indicators (income, education) slightly attenuates this association. Fathers with lowest personality scores show a 38% (SM) and 34% (ES) higher risk of experiencing cohabitation dissolution, compared to the reference group (score 3, all covariates fixed). These risks decrease in scores 2 and 3. A slightly increasing trend for the highest score is indicated. For instance, males with an SM score of 5 show a 7.4% higher separation risk compared to the reference (score 3), but these differences are not statistically significant. Trends are similar in brother comparisons for SM, but ES does not show clear patterns, and statistical uncertainty is comparatively high (Tables 12 and 13).

### Robustness Checks

Several robustness checks have been run. First, different age thresholds (45 and 50) were tested for both marriage (Figs. 10, 11) and fertility analyses (Figs. 12, 13, 14, 15). Patterns do not change but statistical power decreases with higher thresholds. Additionally, previous research suggests that personality-marriage associations may change across birth cohorts, e.g., the positive relationship between social competences and marriage has been found among older cohorts of German men but not younger ones (Lundberg, 2012). However, additional analyses on Swedish cohorts have not shown these patterns for the present study (Figs. 16, 17). This may indicate that the personality-marriage association differs across countries (Germany vs. Sweden). However, both the German and the current study also differ conceptually from each other. The German study measures personality via self-reports on the Big Five several years after a potential marriage, whereas the present study measures personality via psychologists’ evaluations on social maturity most often several years before the potential marriage. Rater-differences or personality changes around marriage could not have been examined that may partially explain the observed differences between Germany and Sweden.

Furthermore, logistic regression models were run for analyses on binary-coded fertility and marriage outcomes. Corresponding coefficients from models with and without SES factors are shown in Figs. 18 and 19. Patterns are consistent with findings from LPM that are shown in Figs. 2 and 4 above. For the manuscript, estimates from LPM are preferred over findings from logistic regression models because coefficients across different models (e.g., with and without SES factors) may be compared based on the first approach, while this is not possible with logistic regression models (Mood, 2010). Average marginal effects from logit models allow such comparisons. However, previous research has shown that LPM coefficients produce similar estimates so that LPM is a reliable model for binary-coded outcomes (Angrist & Pischke, 2009). Additionally, models including both personality factors have been conducted. Magnitudes decrease when controlled for both SM and ES but general patterns persist (Figs. 20, 21, 22, 23, 24).

## Discussion

### Conclusions

This study has examined the prospective association between personality factors from younger ages (SM and ES) and family formation processes by mid-adulthood among males using Swedish register data. As expected, the analyses reveal that SM and ES are positively related to the probability of having ever been married and the number of children by age 39 and higher, and patterns are robust in brother comparisons.

Therefore, personality factors matter for family formation processes, with generally higher magnitudes for SM than ES, and effect sizes are considerable, as demonstrated by comparisons to effect sizes of covariates from the same models. For instance, marriage models without SES indicators produce estimates ranging from − 0.15 (score 1, reference: score 3) to 0.14 (score 5) for SM. These effects are larger than the ones of cognitive skills (from − 0.07 to 0.11) and of birth year (from − 0.05 to 0.09). Moreover, the range is larger than for birth order (− 0.18 to 0.00) and sibling group size (0.00–0.22). Models including SES factors reveal SM coefficients between − 0.06 and 0.09. These are comparable with educational gradients, from which only the doctoral degree shows stronger associations with marriage (coefficient: 0.13). However, income estimates are typically larger than personality magnitudes (between − 0.23 and 0.13). Similar conclusions emerge from models on ES and all fertility analyses. Divorce and cohabitation dissolution are strongly shaped by personality. For instance, the lowest score (1) is linked with a 58% (SM) and 48% (ES) higher risk of getting divorced (reference: score 3). Larger magnitudes can only be detected in the lowest incomes (decile 1: 68% higher risk). Therefore, personality effects are considerable and can compete to some extent with SES effects. This underlines the relevance of personality factors for important life outcomes, aligning previous research (Roberts et al., 2007). This study contributes to the challenge of demographic research to unfold the potential of personality for family formation.

The findings of this study suggest that education and income mediate the association between personality and family formation, given that the coefficients between models with and without SES factors diminish. However, further mediation analyses are required for reliable conclusions on mediation. There may also be arguments for rather small mediation effects. Among others, the temporal sequence of mediators and family formation outcomes might be reversed for many individuals in the current study. For instance, the majority of children have been born outside of marriage in contemporary Sweden (Eurostat, 2018), i.e., marriage may not necessarily mediate the personality-fertility link. Similarly, income may increase after becoming a parent, rather than the other way around. According to previous research, particularly men benefit from income increases after entering parenthood, known as the "fatherhood premium effect" (Angelov et al., 2016; Bygren et al., 2021).

Additionally, findings of this study indicate that lower scores on both SM and ES predict higher divorce risks by mid-adulthood, with no clear patterns among higher personality scores. These findings are partly in line with expectations that are based on mixed evidence from previous studies (Bleidorn et al., 2018; Spikic & Mortelmans, 2021). For instance, social competences are linked with higher infidelity risks (Spikic & Mortelmans, 2021), indicating lower union stability. Contrary, social personality indicators are also associated with higher life satisfaction after getting married (Boyce et al., 2016), suggesting higher marriage stability. Therefore, the relationship between social competences and marital behavior appears to be complicated. Moreover, previous findings indicate that males become more introvert, i.e., social competences decrease, and more emotionally stable after marriage, which also affects marital satisfaction (Lavner et al., 2018), and therefore marital stability. However, the data do not allow examination for potential changes in SM or ES. Previous evidence further suggests that associations between divorce and social personality indicators (positive) as well as ES (negative) are relatively robust across marriage cohorts (Boertien & Mortelmans, 2018).

Similar patterns emerge regarding cohabitation dissolution, i.e., low scores on both personality factors are linked with higher separation risks. Trends persist in within-family comparisons but with larger statistical uncertainty. Cohabitation analyses are restricted to couples who live in the same geographical unit and have a child together. This is a strong restriction due to two reasons: first, cohabitation has become a more common type of union in Sweden (Duvander & Kridahl, 2020) and childless cohabiters are neglected in this approach. Second, the presence of children is one of the strongest barriers to dissolution (Knoester & Booth, 2000; Todesco, 2011). Therefore, non-parents may face higher dissolution risks and a greater relevance of psychological factors for their relationship stability than parents.

However, it remains speculative to which extent the personality-dissolution association is different among non-parents. For instance, men with low SM scores are characterized by, among others, low responsibility, and a child may confront such men with tasks that they do not feel ready for, which may lead to them ending the relationship. On the other hand, a joint child may be considered a barrier to dissolution, even among fathers with low SM, which may encourage them to stay with the partner. Men with high levels of SM, in turn, may be less likely to experience union dissolution when a child was born, as they may take the responsibility of childcare.

**Fig. 9:**
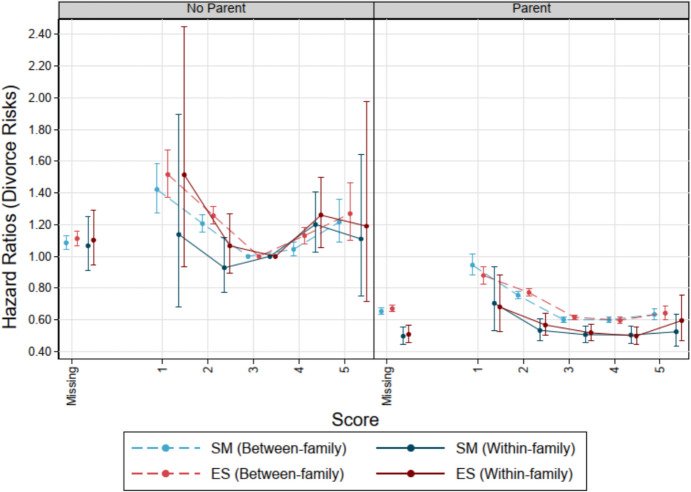
The relationship between personality factors measured at ages 17–20 and divorce risks by age 39 and higher among Swedish men born 1963–1979. Cox Proportional Hazards models by parental status, error bars are 95% confidence intervals. *Note* Models control for cognitive abilities, income, education, birth year, birth order, and in case of between-family considerations for sibling group size

**Fig. 10:**
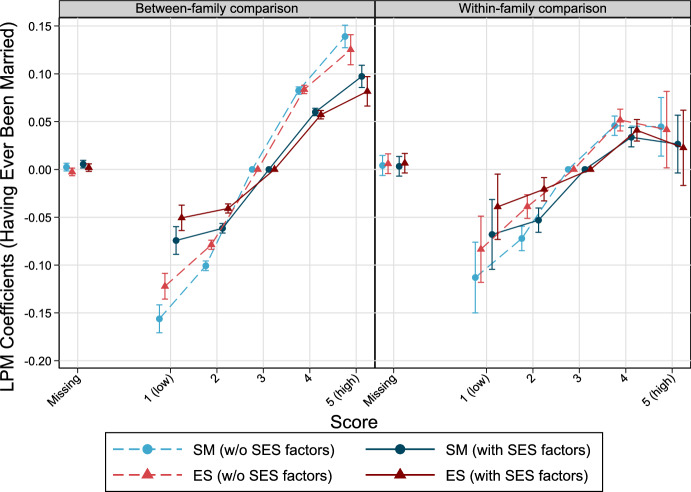
The relationship between personality factors measured at ages 17–20 and the probability to get married by age 45 and higher among Swedish men born 1963–1979. Linear probability models, error bars are 95% confidence intervals. *Note* Models without SES factors control for cognitive abilities, birth year, birth order, and in case of between-family considerations for sibling group size. Models with SES factors include income and education, additionally

**Fig. 11:**
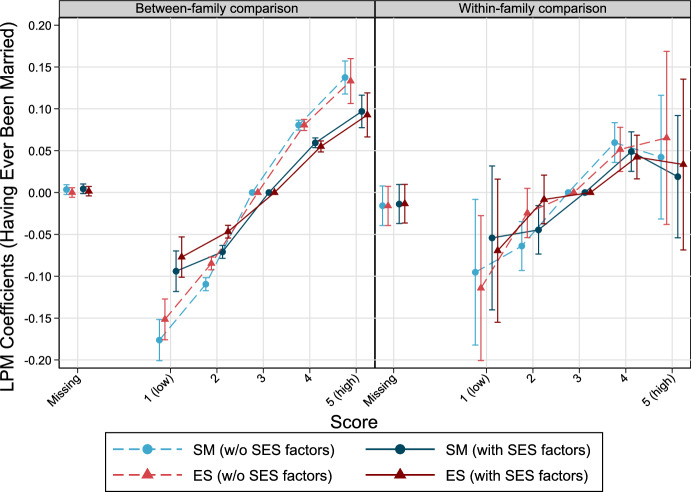
The relationship between personality factors measured at ages 17–20 and the probability to get married by age 50 and higher among Swedish men born 1963–1979. Linear probability models, error bars are 95% confidence intervals. *Note* Models without SES factors control for cognitive abilities, birth year, birth order, and in case of between-family considerations for sibling group size. Models with SES factors include income and education, additionally

**Fig. 12:**
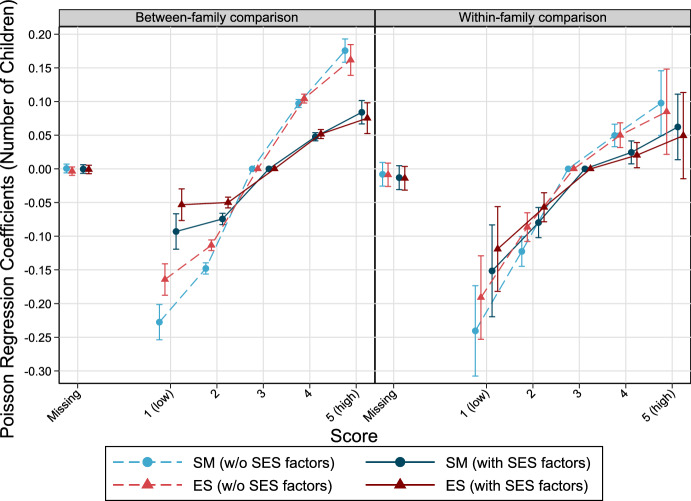
The relationship between personality factors measured at ages 17–20 and number of children by age 45 and higher among Swedish men born 1963–1979. Poisson regression models, error bars are 95% confidence intervals. *Note* Models without SES factors control for cognitive abilities, birth year, birth order, and in case of between-family considerations for sibling group size. Models with SES factors include income and education, additionally

**Fig. 13:**
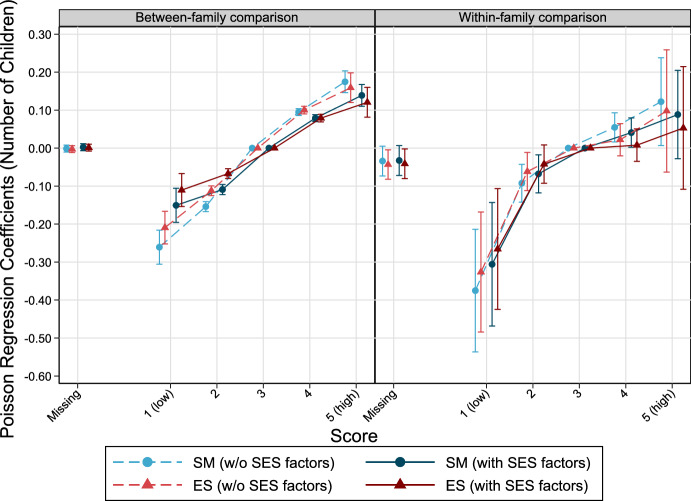
The relationship between personality factors measured at ages 17–20 and number of children by age 50 and higher among Swedish men born 1963–1979. Poisson regression models, error bars are 95% confidence intervals. *Note* Models without SES factors control for cognitive abilities, birth year, birth order, and in case of between-family considerations for sibling group size. Models with SES factors include income and education, additionally

**Fig. 14:**
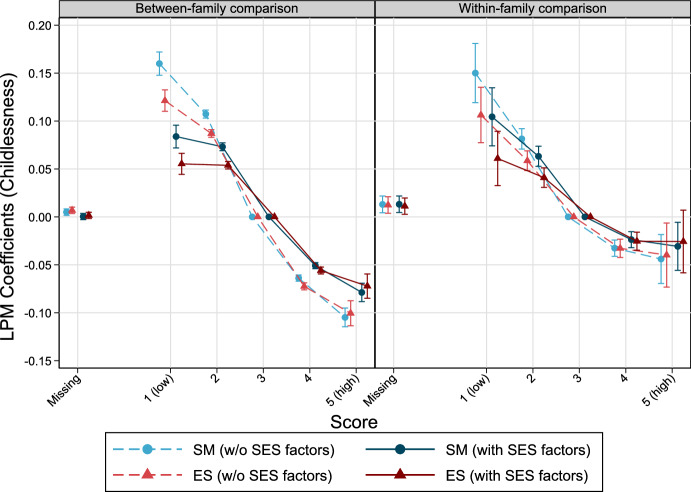
The relationship between personality factors measured at ages 17–20 and childlessness by age 45 and higher among Swedish men born 1963–1979. Linear probability models, error bars are 95% confidence intervals. *Note* Models without SES factors control for cognitive abilities, birth year, birth order, and in case of between-family considerations for sibling group size. Models with SES factors include income and education, additionally

**Fig. 15:**
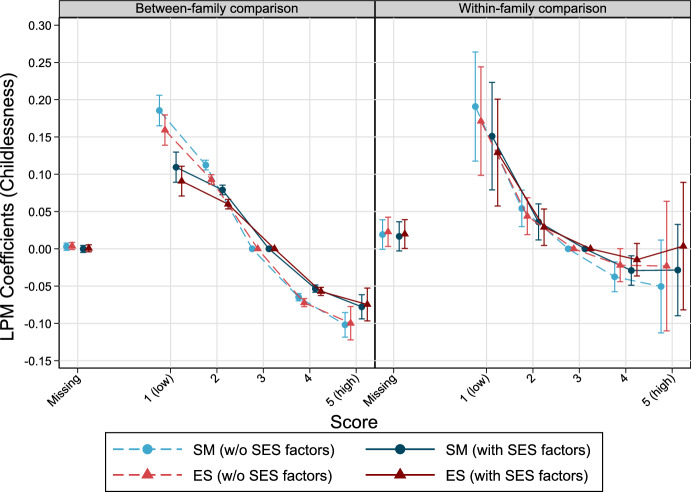
The relationship between personality factors measured at ages 17–20 and childlessness by age 50 and higher among Swedish men born 1963–1979. Linear probability models, error bars are 95% confidence intervals. *Note* Models without SES factors control for cognitive abilities, birth year, birth order, and in case of between-family considerations for sibling group size. Models with SES factors include income and education, additionally

**Fig. 16:**
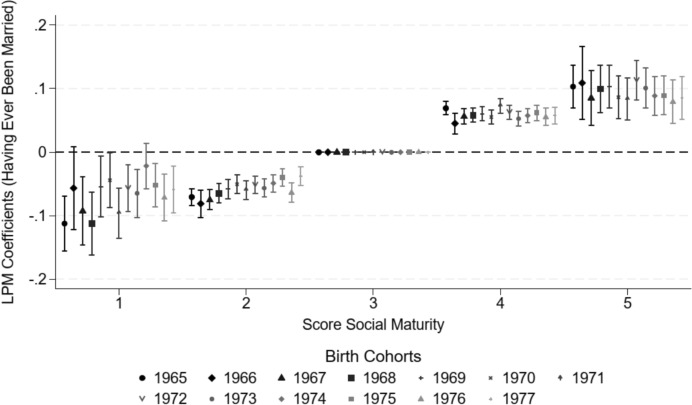
Coefficients of social maturity scores measured at ages 17–20 on marital status by age 39 and higher among Swedish men born 1965–1977 across birth cohorts. Linear probability models, error bars are 95% confidence intervals. *Note* Models control for cognitive abilities, birth order, sibling group size, income and education. Birth cohorts 1963, 1964, 1978, and 1979 are not shown due to high statistical uncertainty (too few cases and too large confidence intervals). Coefficients from the category of missing values are not shown for the same reason

**Fig. 17:**
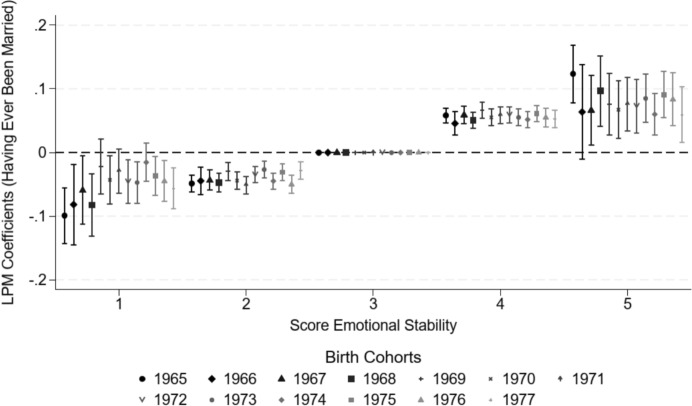
Coefficients of emotional stability scores measured at ages 17–20 on marital status by age 39 and higher among Swedish men born 1965–1977 across birth cohorts. Linear probability models, error bars are 95% confidence intervals. *Note* Models control for cognitive abilities, birth order, sibling group size, income and education. Birth cohorts 1963, 1964, 1978, and 1979 are not shown due to high statistical uncertainty (too few cases and too large confidence intervals). Coefficients from the category of missing values are not shown for the same reason

**Fig. 18:**
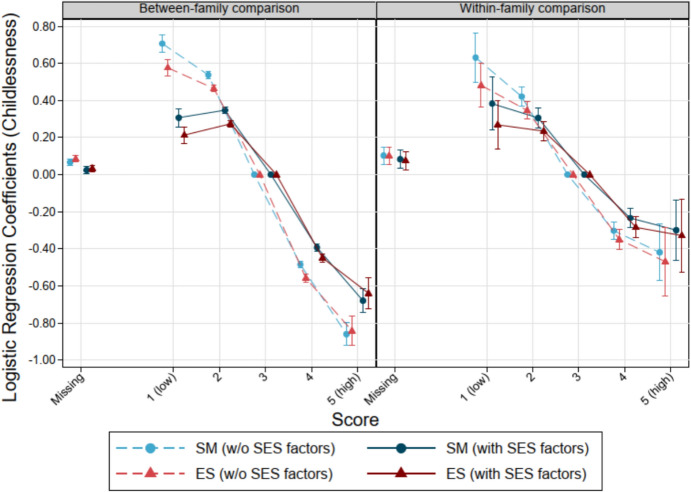
The relationship between personality factors measured at ages 17–20 and childlessness by age 39 and higher among Swedish men born 1963–1979. Logistic regression models, error bars are 95% confidence intervals. *Note* Models without SES factors control for cognitive abilities, birth year, birth order, and in case of between-family considerations for sibling group size. Models with SES factors include income and education, additionally

**Fig. 19:**
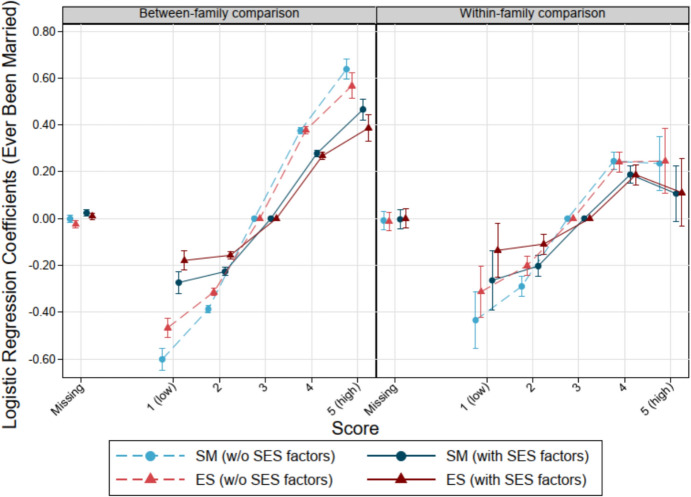
The relationship between personality factors measured at ages 17–20 and the probability to get married by age 39 and higher among Swedish men born 1963–1979. Logistic regression models, error bars are 95% confidence intervals. *Note* Models without SES factors control for cognitive abilities, birth year, birth order, and in case of between-family considerations for sibling group size. Models with SES factors include income and education, additionally

**Fig. 20:**
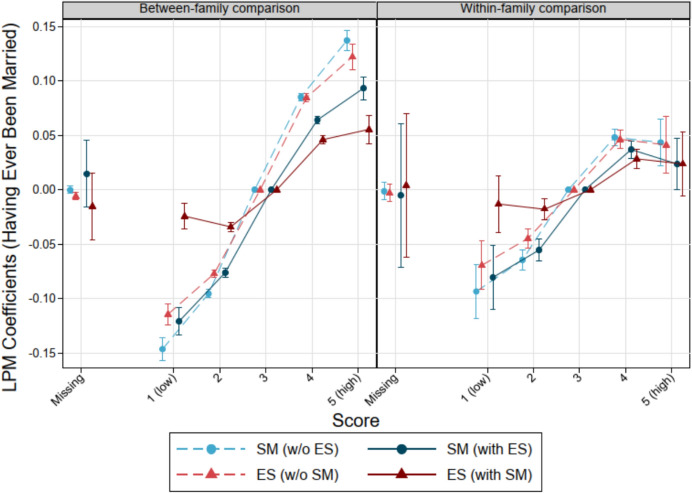
The relationship between personality factors measured at ages 17–20 and the probability to get married by age 39 and higher among Swedish men born 1963–1979 (both personality factors). Linear probability models, error bars are 95% confidence intervals. *Note* Models without the other personality factor control for cognitive abilities, birth year, birth order, and in case of between-family considerations for sibling group size. Models with the other personality factor include SM or ES, additionally

**Fig. 21:**
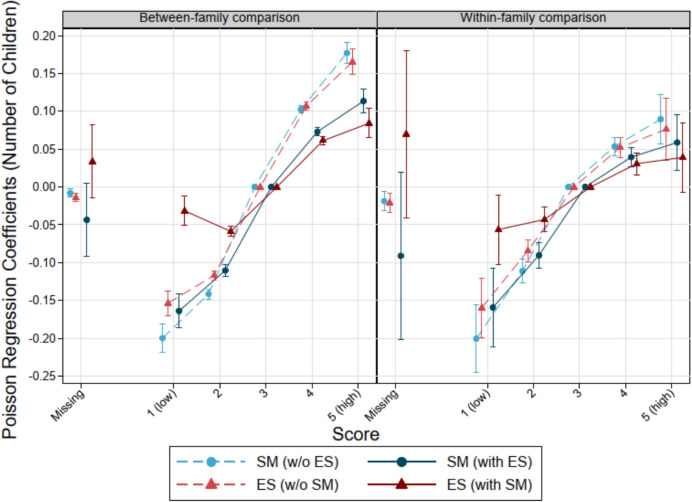
The relationship between personality factors measured at ages 17–20 and number of children by age 39 and higher among Swedish men born 1963–1979 (both personality factors). Poisson regression models, error bars are 95% confidence intervals. *Note* Models without the other personality factor control for cognitive abilities, birth year, birth order, and in case of between-family considerations for sibling group size. Models with the other personality factor include SM or ES, additionally

**Fig. 22:**
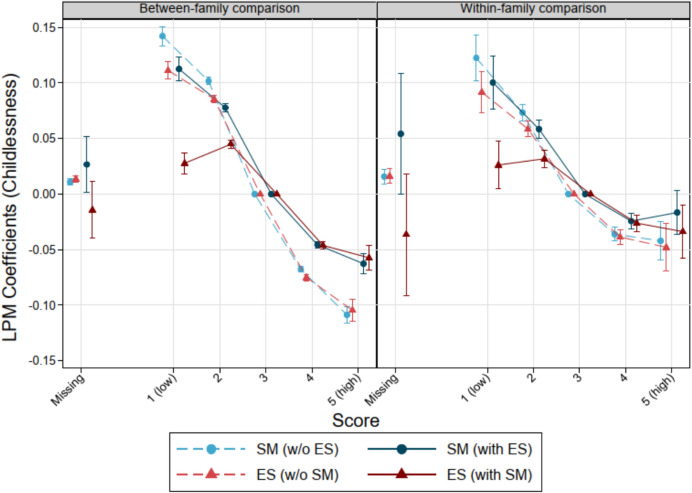
The relationship between personality factors measured at ages 17–20 and childlessness by age 39 and higher among Swedish men born 1963–1979 (both personality factors). Linear probability models, error bars are 95% confidence intervals. Note: Models without the other personality factor control for cognitive abilities, birth year, birth order, and in case of between-family considerations for sibling group size. Models with the other personality factor include SM or ES, additionally

**Fig. 23:**
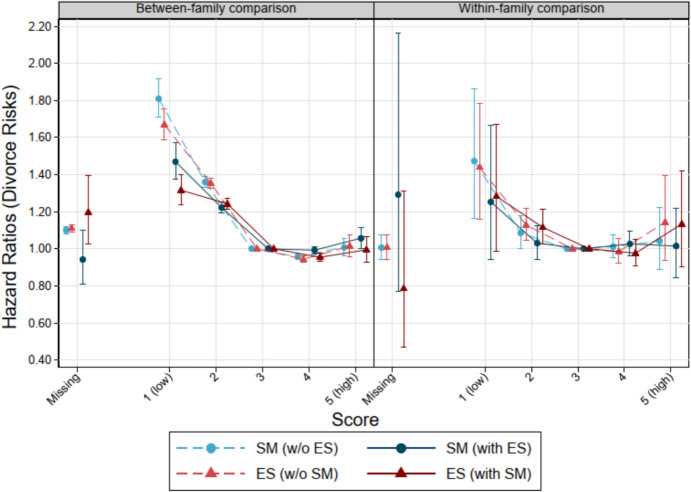
The relationship between personality factors measured at ages 17–20 and divorce risks by age 39 and higher among Swedish men born 1963–1979 (both personality factors). Cox Proportional Hazards models, error bars are 95% confidence intervals. *Note* Models without the other personality factor control for cognitive abilities, birth year, birth order, and in case of between-family considerations for sibling group size. Models with the other personality factor include SM or ES, additionally

**Fig. 24:**
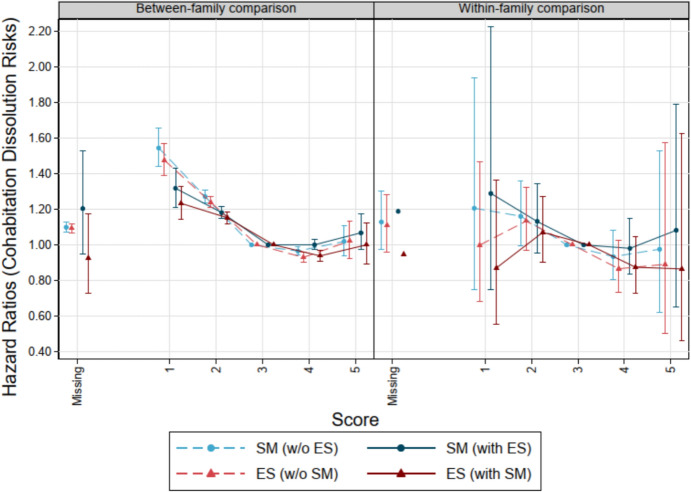
The relationship between personality factors measured at ages 17–20 and cohabitation dissolution risks by age 39 and higher among Swedish men born 1963–1979 (both personality factors). Cox Proportional Hazards models, error bars are 95% confidence intervals. *Note* Models without the other personality factor control for cognitive abilities, birth year, birth order, and in case of between-family considerations for sibling group size. Models with the other personality factor include SM or ES, additionally. For visualization purposes, not all confidence intervals are shown in within-family comparisons

Similarly, ES may show different associations with dissolution risks among childless men compared to fathers. Childless males with low ES scores may be more likely to separate from a partner than fathers with low ES scores since fathers may consider having a child a stabilizing factor for the relationship. On the other hand, a child may be considered an additional burden and resource of stress that men with low ES scores may refrain from, indicating higher dissolution risks. Additional analyses in the present study based on divorce risks reveal no large differences in patterns by parental status, although divorce risks among non-parents are generally higher (Fig. 9). Lower personality scores among both parents and non-parents are associated with higher divorce risks, whereas the patterns flatten among higher scores of the parents, high personality factor scores among non-parents are also linked with higher divorce-risks, though. However, future studies are needed in order to identify differences in dissolution risks between cohabiting couples with and without children.

### Strengths and Limitations

This study has certain limitations. For instance, no personality information has been available for women although previous research has suggested gender differences in the link between personality and family formation (Jokela et al., 2011; Peters, 2023). Including women in the analyses would also allow to examine couples’ behavior. Recent research has shown that large social personality differences between partners increase dissolution risks, in particular among lower educated groups (Hofmann & Krapf, 2024). These associations may further be examined, and even extended to marital and fertility behavior.

Additionally, there is a non-neglectable proportion of missing values for the personality measures (SM: 19.58%; ES: 19.65%), which is still included in the analyses. It remains unclear why one fifth of the men has not been assessed on the personality traits. Additional descriptive analyses show that more than 99% of the men with missing value on one personality factor have not received an evaluation on the other either (Table 14). Furthermore, 26.06% of the males with missing values on leadership skills were not evaluated on personality factors but males were assessed on their personality scores throughout all IQ categories (Table 15). Large proportions of men with low hearing test scores (around 50%) as well as missing values on hearing (24.28%) and technical skills tests (28.94%) completed the conscription without personality assessments (Table 16). This may be linked with the selection of potential candidates for all military, but especially officer positions who need to show certain levels of SM, ES, and leadership skills (Ludvigsson et al., 2022). Males with missing SM and ES values do not differ substantially regarding marriage or fertility probabilities from the reference group (score 3), but they show higher separation and divorce risks.

Furthermore, the purposes of measuring personality may differ between the military and demographic or psychological surveys. The measures from this study come from interviews, and these usually produce less stable personality levels than self-reports from questionnaires (Hopwood & Bleidorn, 2018). This may be particularly problematic since personality may change after certain life events (e.g., the transition to first romantic relationship or from school to work/college) (Bleidorn et al., 2018), or when longer time periods are considered (Harris et al., 2016; Hopwood & Bleidorn, 2018). However, other studies indicate a relatively high stability of personality over the life course (Damian et al., 2015; Lucas & Donnellan, 2011), and life events do not lead to large personality changes (Costa Jr. et al., 2000; Neyer & Asendorpf, 2001), in particular not in the long-term (Allemand et al., 2015). Although the degree of stability in ES and SM from this data over time is uncertain, the prospective design avoids any reverse causality, at least.

On the other hand, this study also reveals strengths. Population-wide administrative register data allow to link personality factors from young ages to demographical events on a population level. Furthermore, sibling comparisons rule out the confounding effects from shared family background factors. Register data face fewer selection problems than surveys since survey participation is voluntary. As one example, disadvantaged males might be less likely to participate in a survey, and to have offspring or to marry. The analyses, however, include the vast majority of Swedish males from birth cohorts 1963–1979—also men who would reject survey participation. Therefore, less bias in the findings compared to studies using survey data can be assumed.

### Implications and Outlook

The findings have a number of practical implications. For instance, high SM and ES scores may be beneficial for union formation resulting in inequalities on the partner market. Males may or may not enter one or more stable partnership unions over the life course. The findings of this study show that males with higher ES and SM scores are more likely to get married suggesting inequalities in partnering processes. This may be partly based on partner preferences. For instance, previous research has shown that females with higher conscientiousness prefer men with higher social factor levels, whereas more extraverted females search for men with lower social skills (Dupuy & Galichon, 2014). This results in men facing difficult trade-offs depending which type of women they would like to attract.

Moreover, partnering inequalities may also affect health outcomes. Singles show higher mortality risks (e.g., Roelfs et al., 2011) and worse well-being (e.g., Wright & Brown, 2017), whereas partnered individuals show better mental health (e.g., Willitts et al., 2004). Therefore, men with lower SM & ES scores (i.e., lower marriage probabilities, higher dissolution risks), may also suffer from worse health outcomes. Similar effects may emerge from findings of fertility analyses of this study.

More research on the prospective association between personality and family formation is needed, in particular since previous research suggests an increasing relevance of psychological factors for family formation processes (Aldén et al., 2022). It will be interesting to examine the link between personality and family formation among women based on observed gender differences in previous studies (Jokela, 2012; Jokela et al., 2011). The personality-family formation link may also be studied in other cultural contexts, in particular regarding the positive association between SES and fertility, which is a specific characteristic of Nordic countries.

## Figures and Tables

**Table 2:**
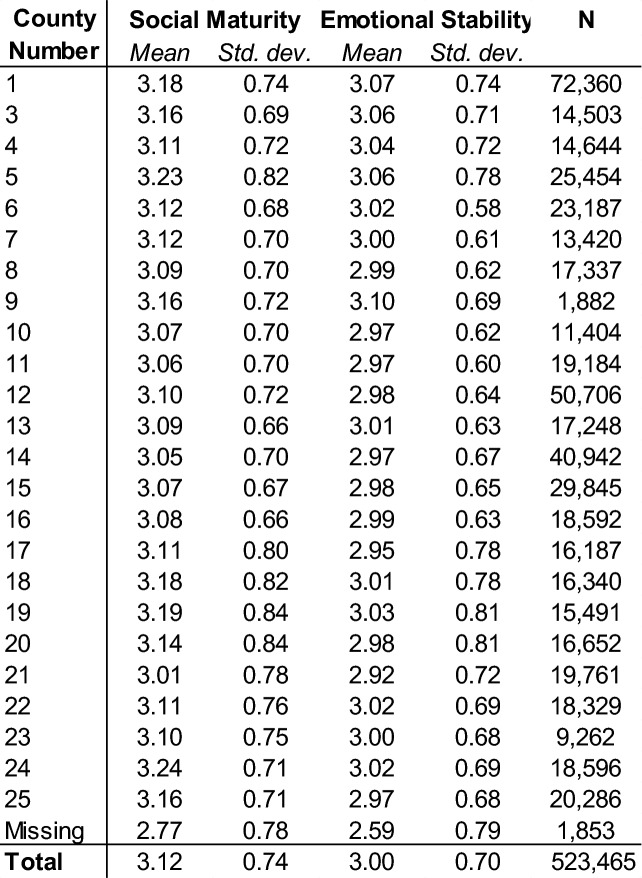
Personality mean values and standard deviations across counties

Missing values on personality factors are excluded here, which reduces the sample size for these bivariate analyses to 523,465 men.

**Table 3:**
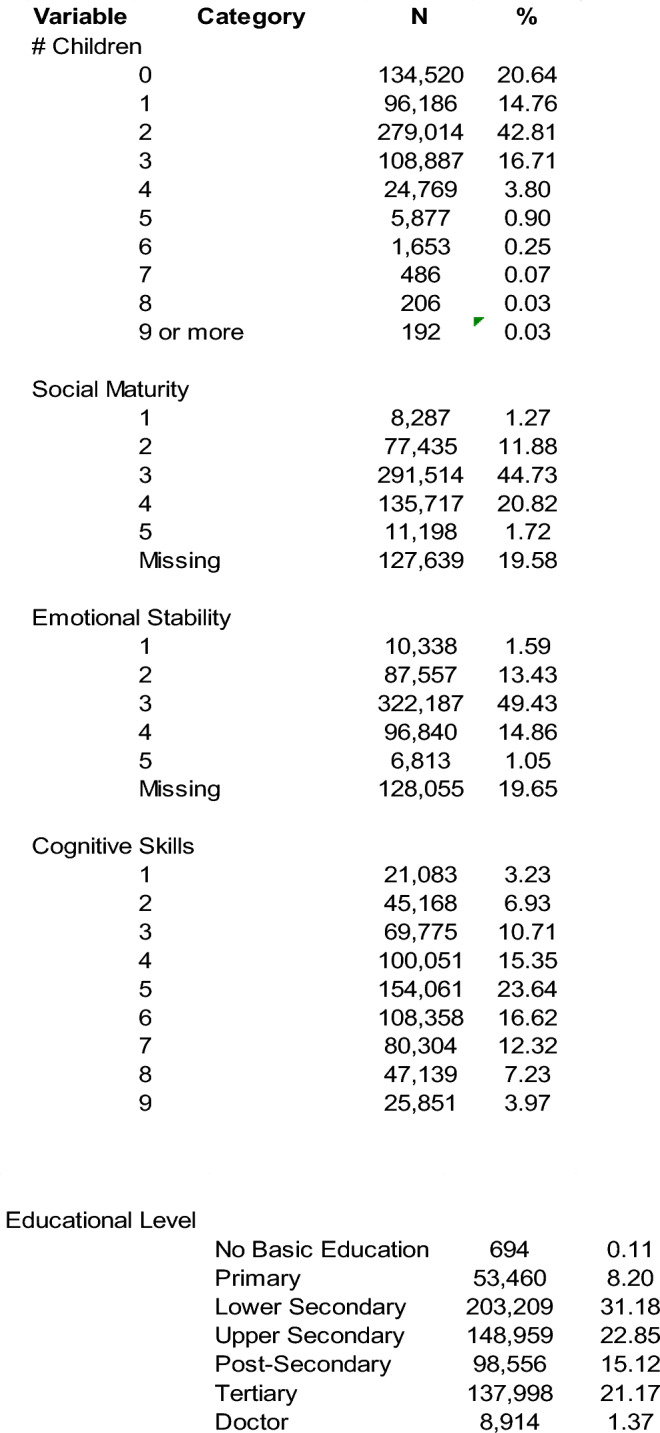

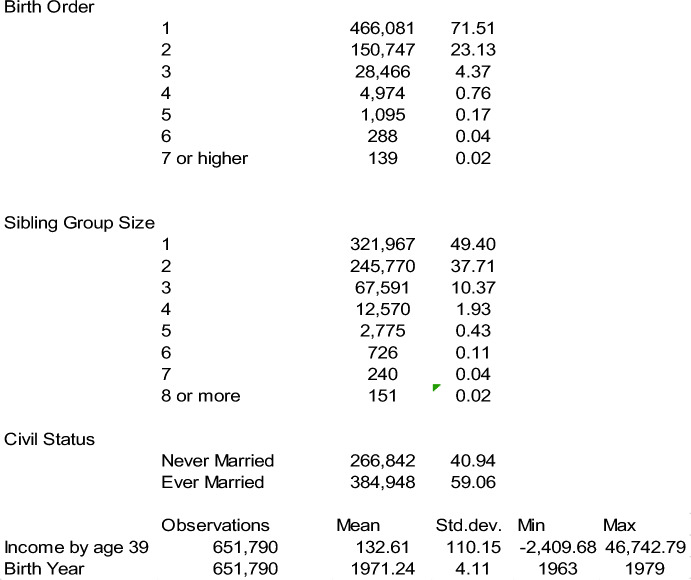
Descriptive statistics—variables

**Table 4:**
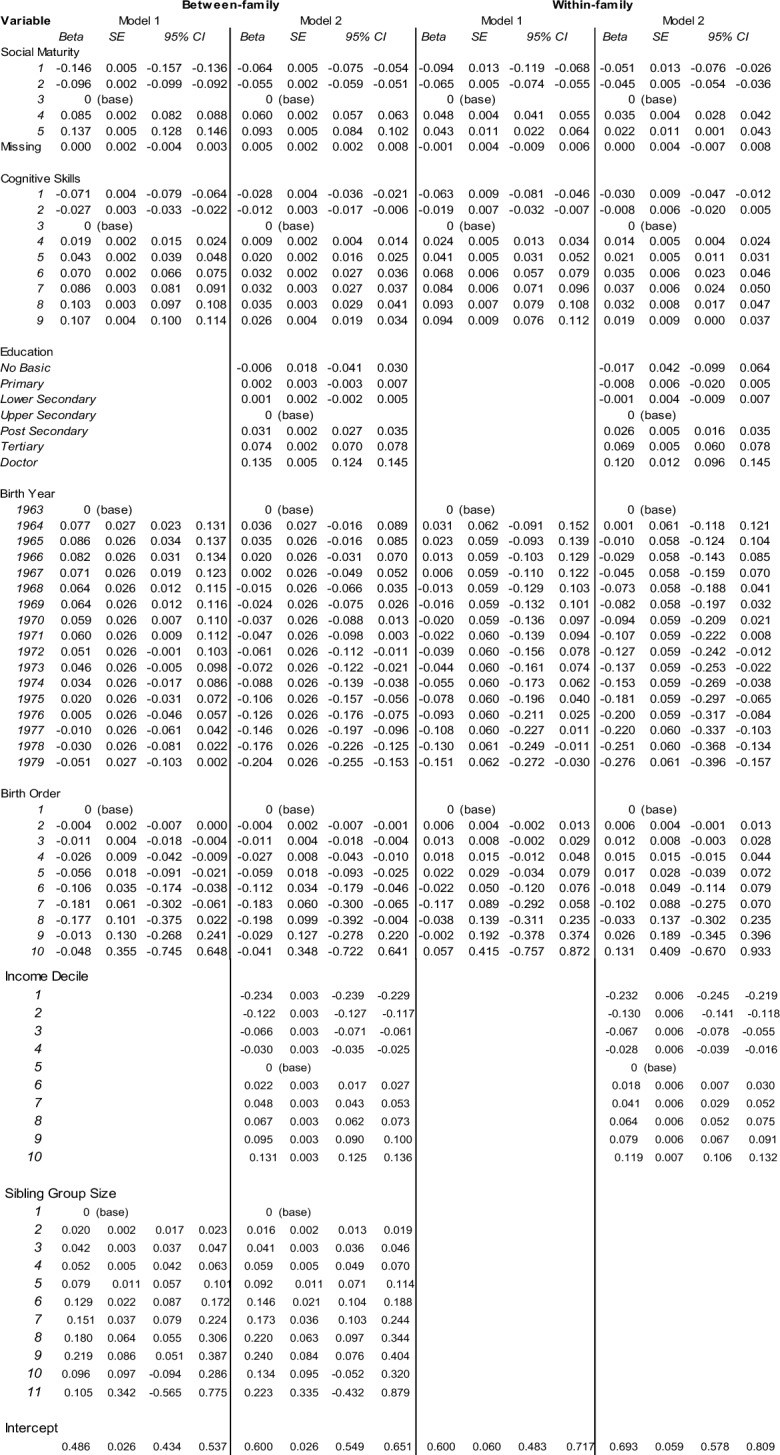
Linear probability model estimates (marriage), social maturity

**Table 5:**
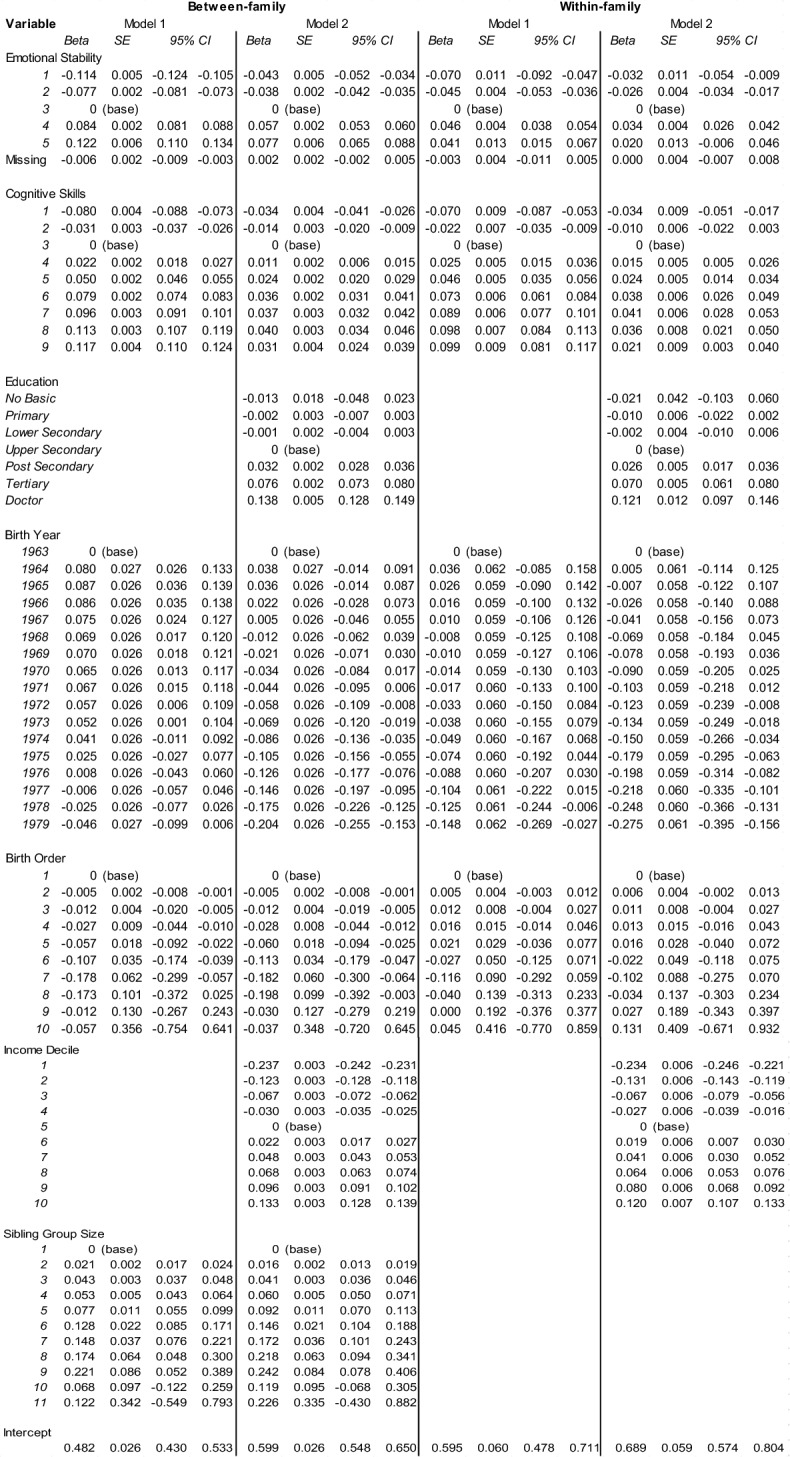
Linear probability model estimates (marriage), emotional stability

**Table 6:**
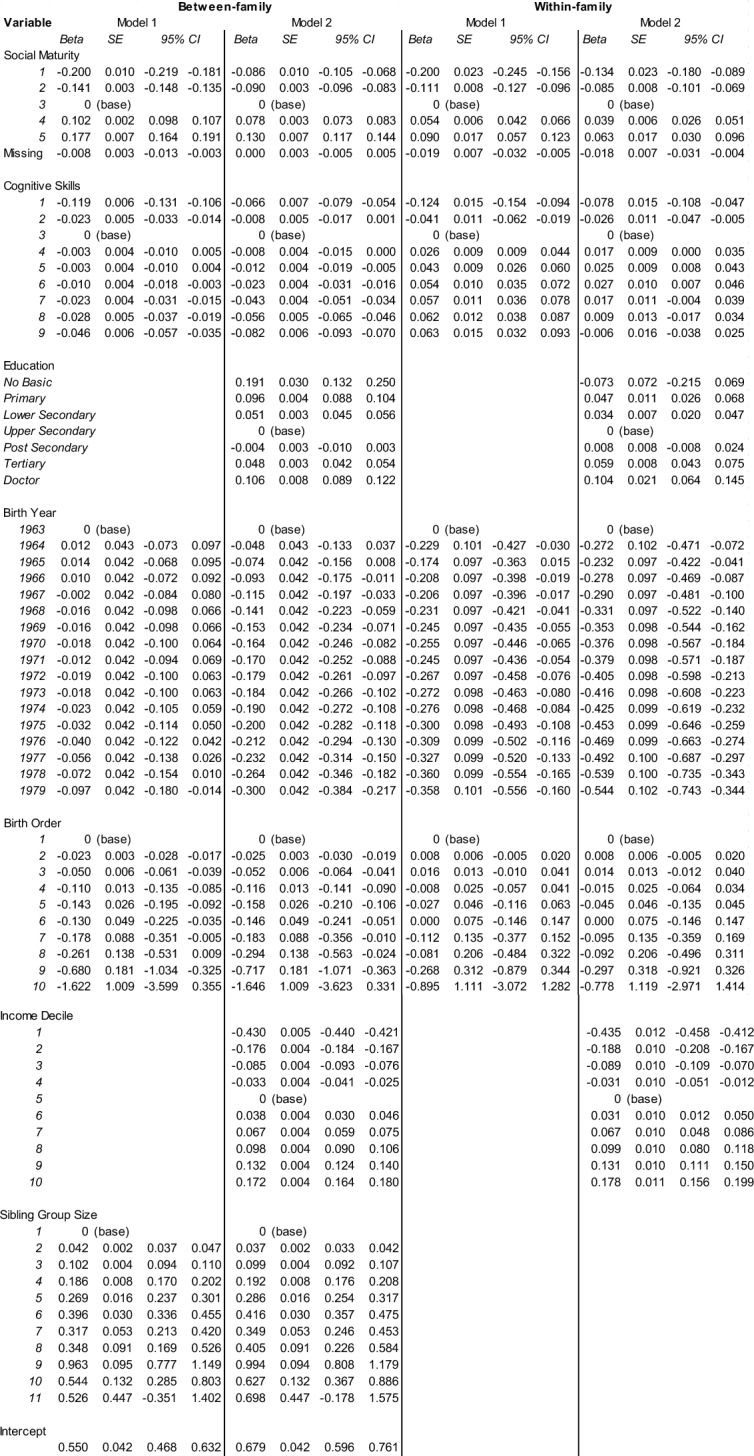
Poisson regression model estimates (number of children), social maturity

**Table 7:**
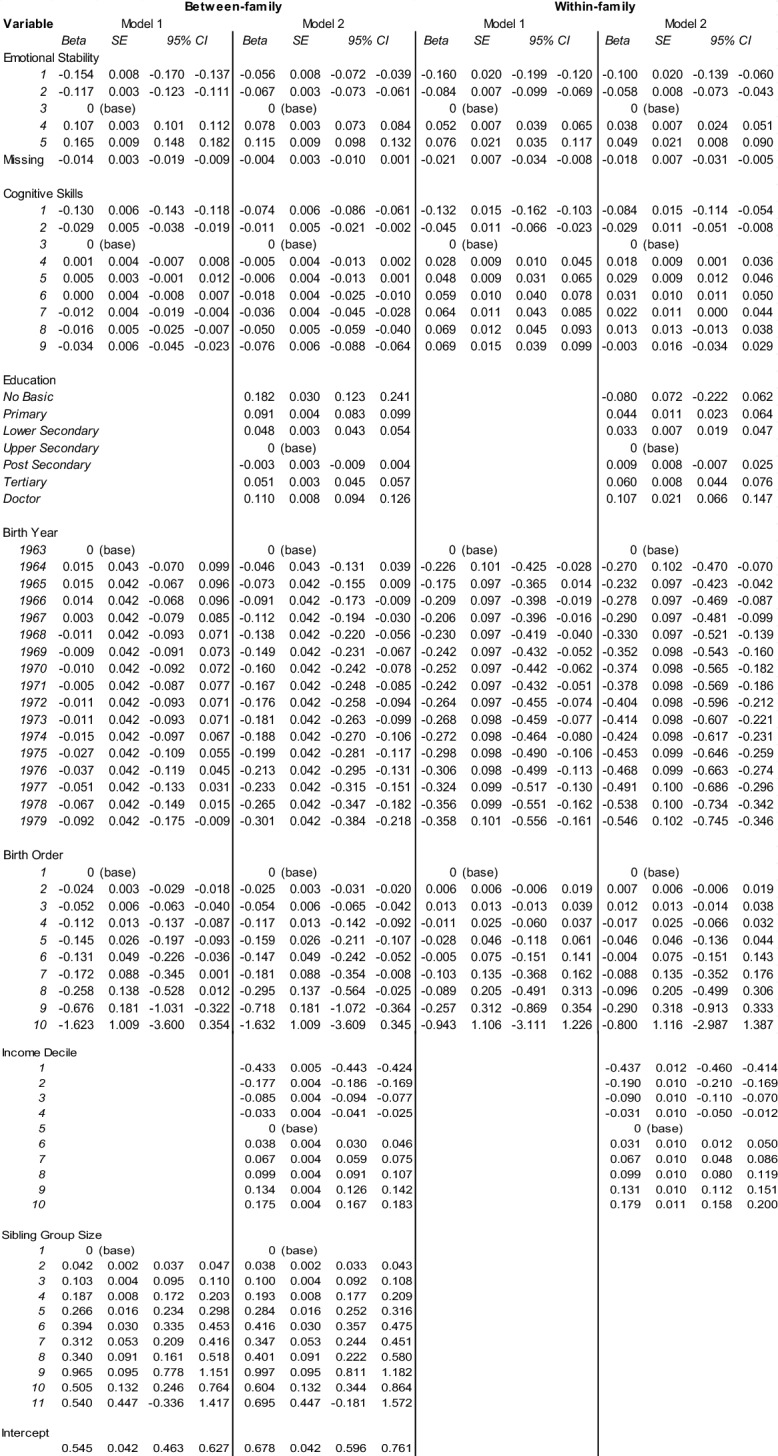
Poisson regression model estimates (number of children), emotional stability

**Table 8:**
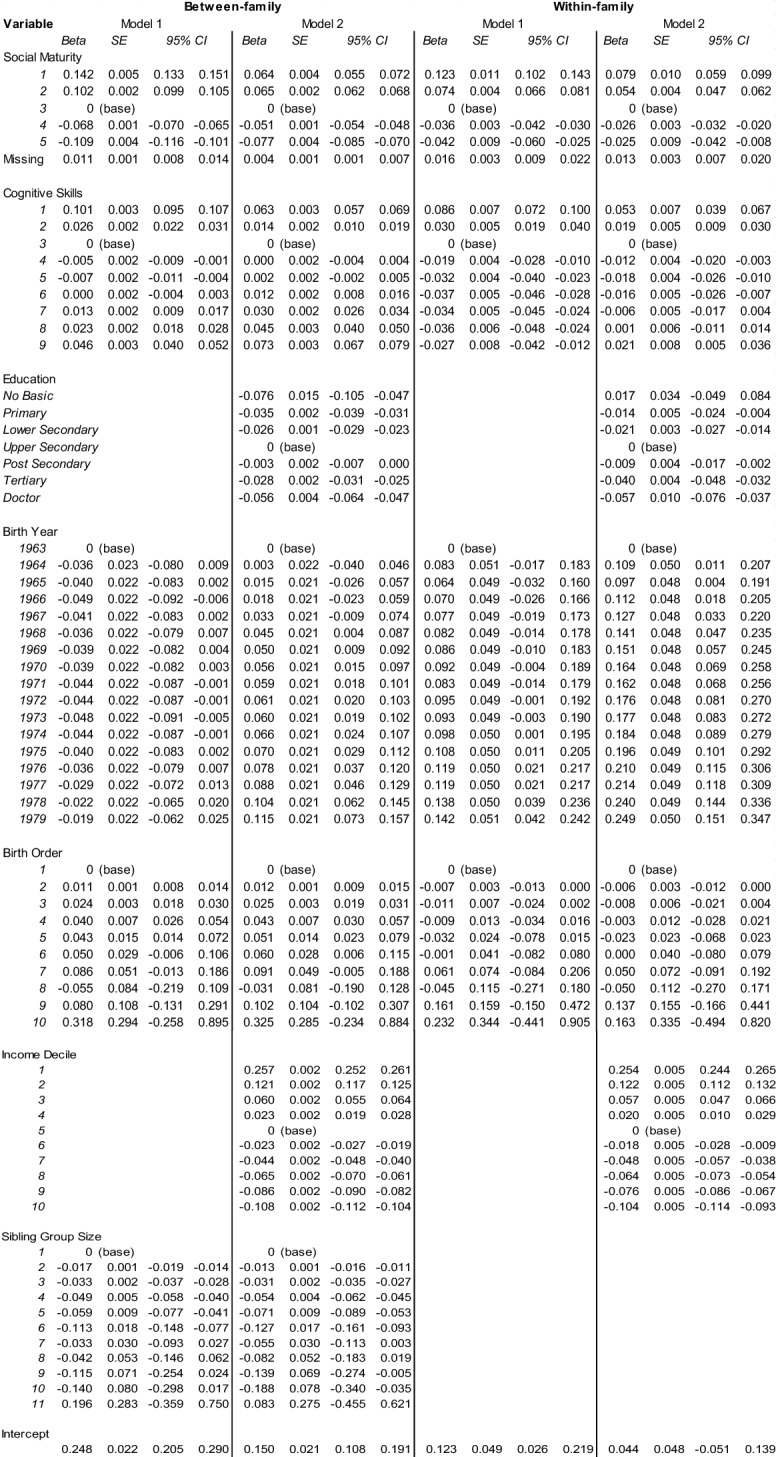
Linear probability model estimates (childlessness), social maturity

**Table 9:**
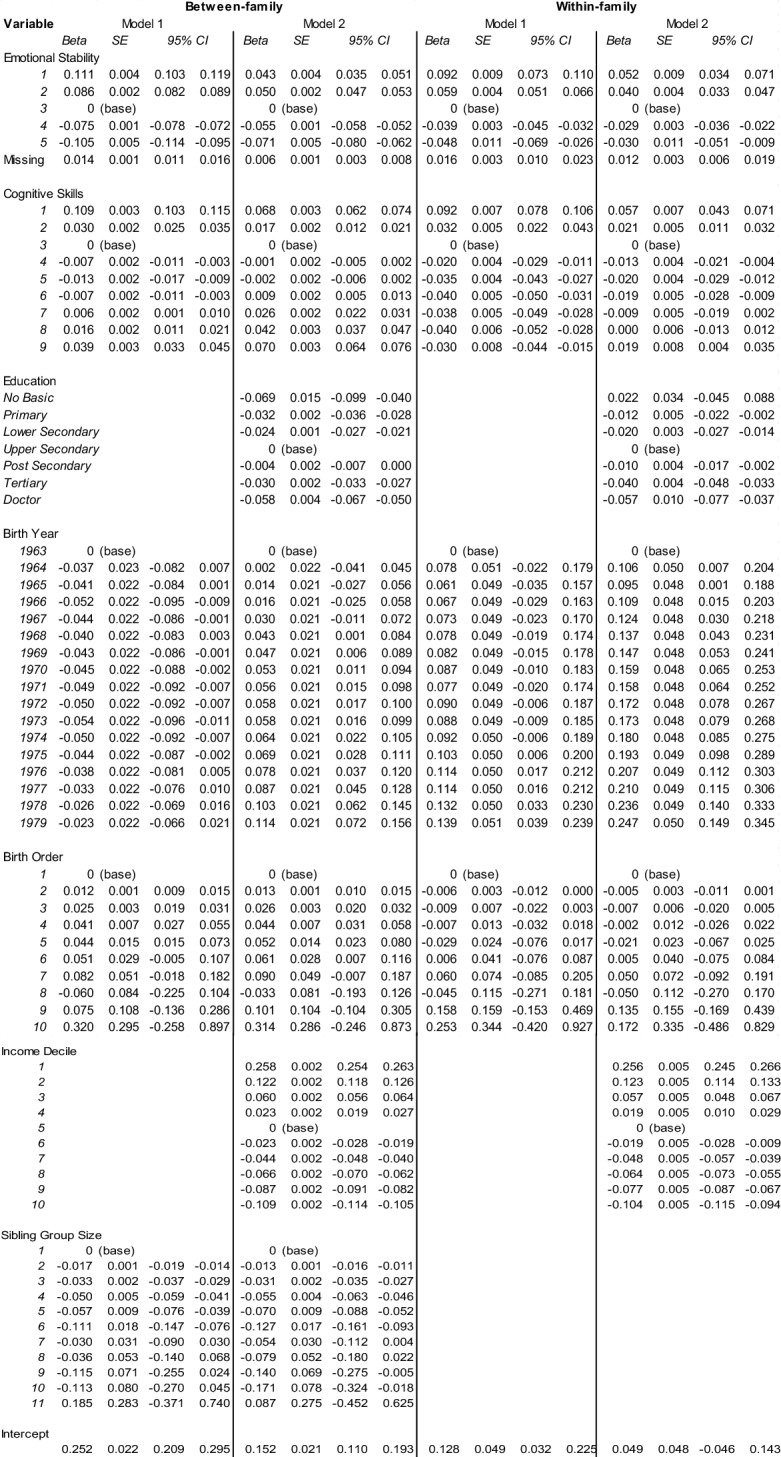
Linear probability model estimates (childlessness), emotional stability

**Table 10:**
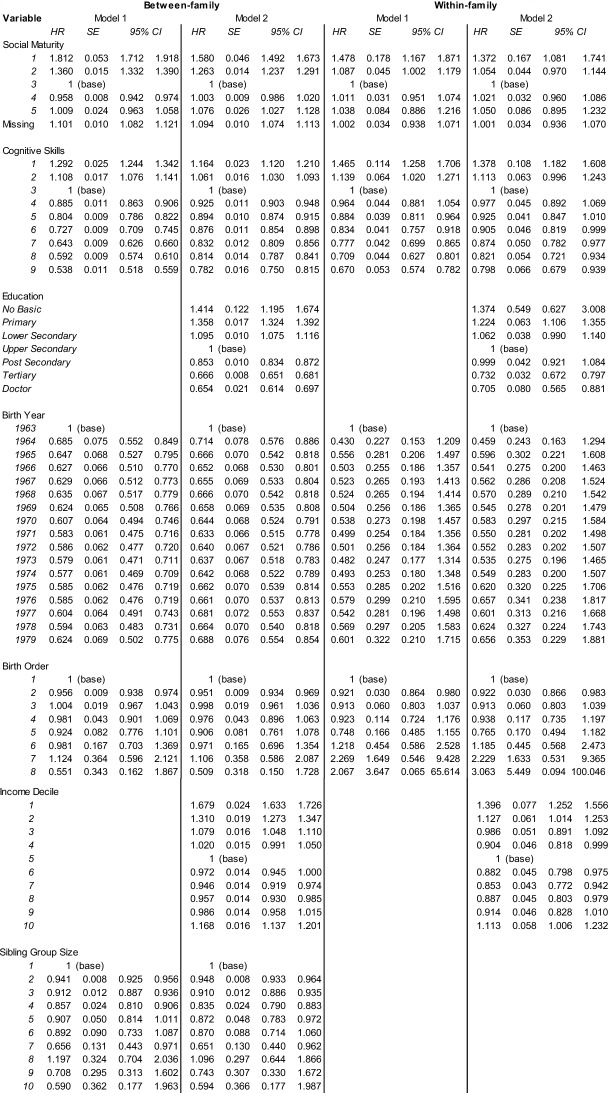
Cox proportional hazards model estimates (divorce), social maturity

**Table 11:**
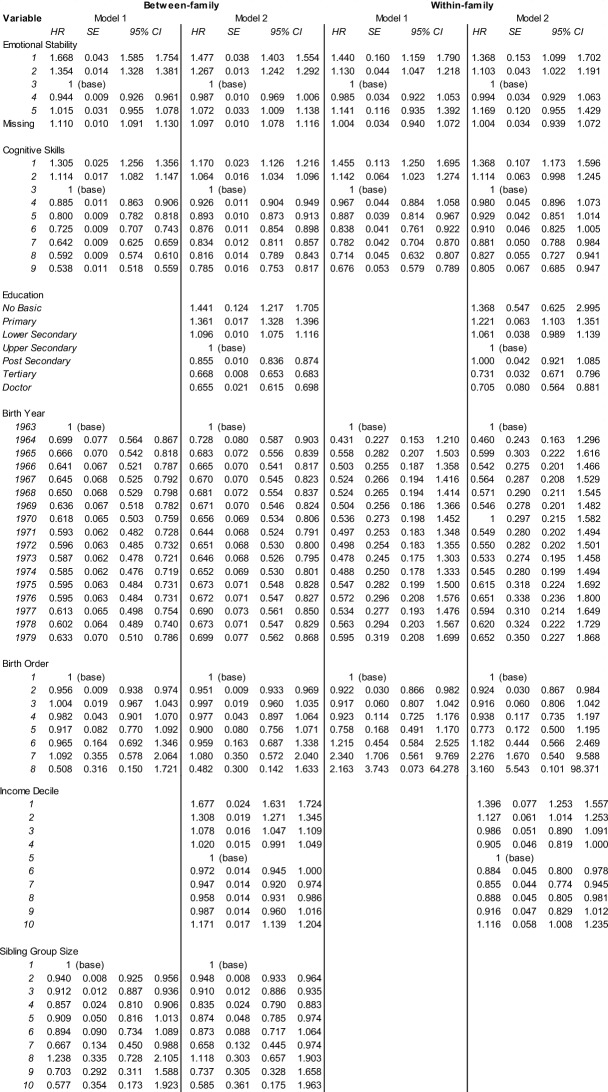
Cox proportional hazards model estimates (divorce), emotional stability

**Table 12:**
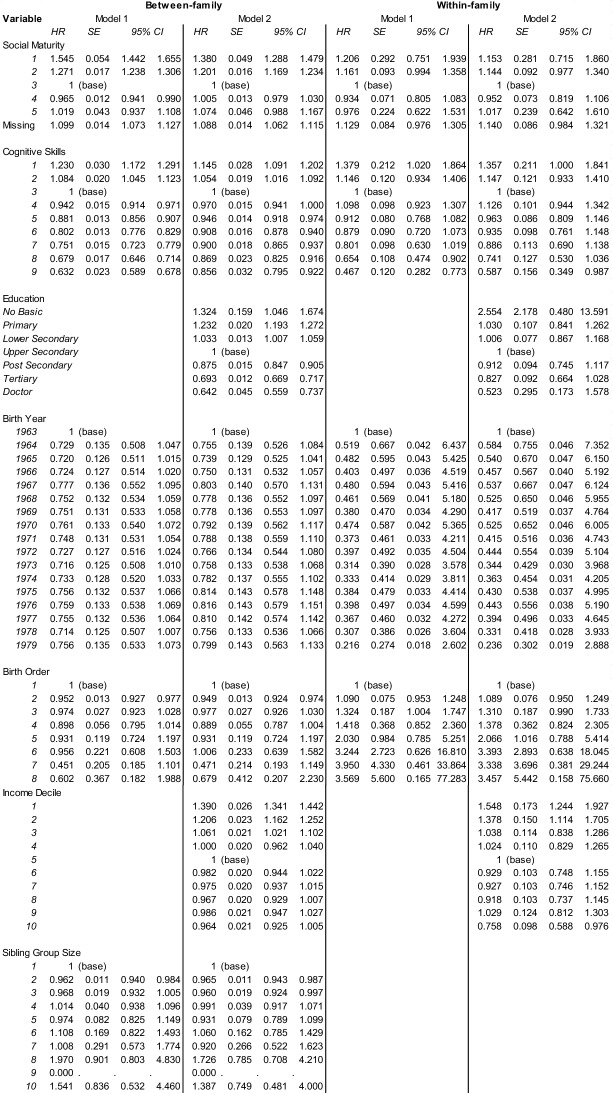
Cox proportional hazards model estimates (cohabitation dissolution), social maturity

**Table 13:**
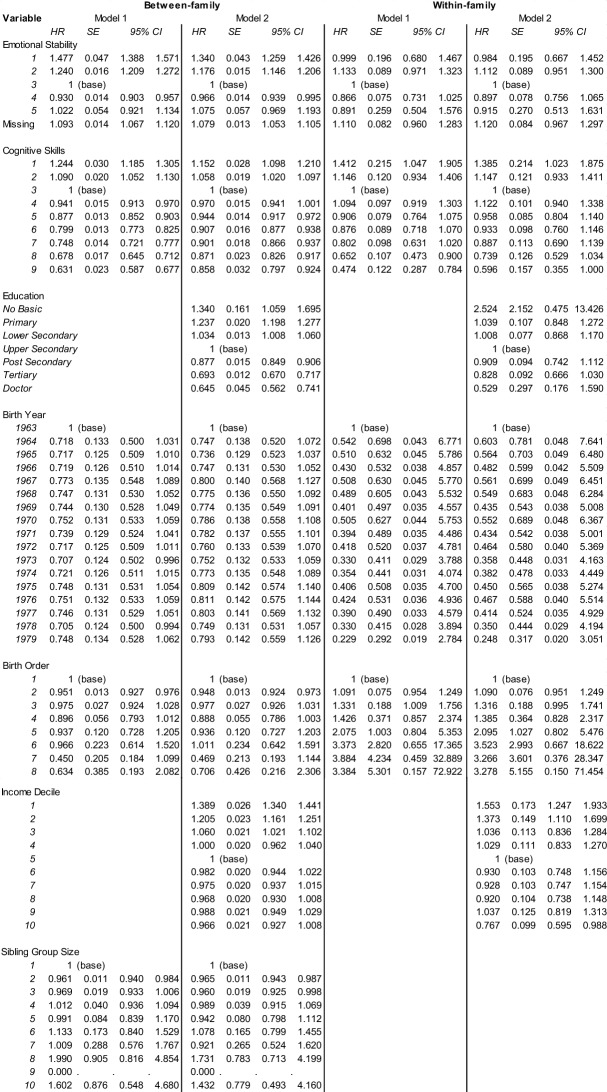
Cox proportional hazards model estimates (cohabitation dissolution), emotional stability

**Table 14:**
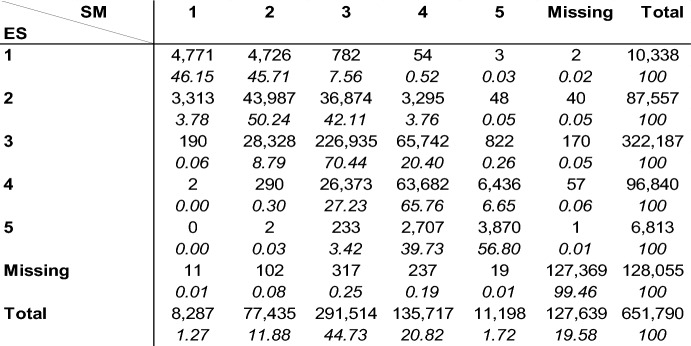
Descriptive statistics, frequency table with social maturity (SM) and emotional stability (ES), row percentages

**Table 15:**
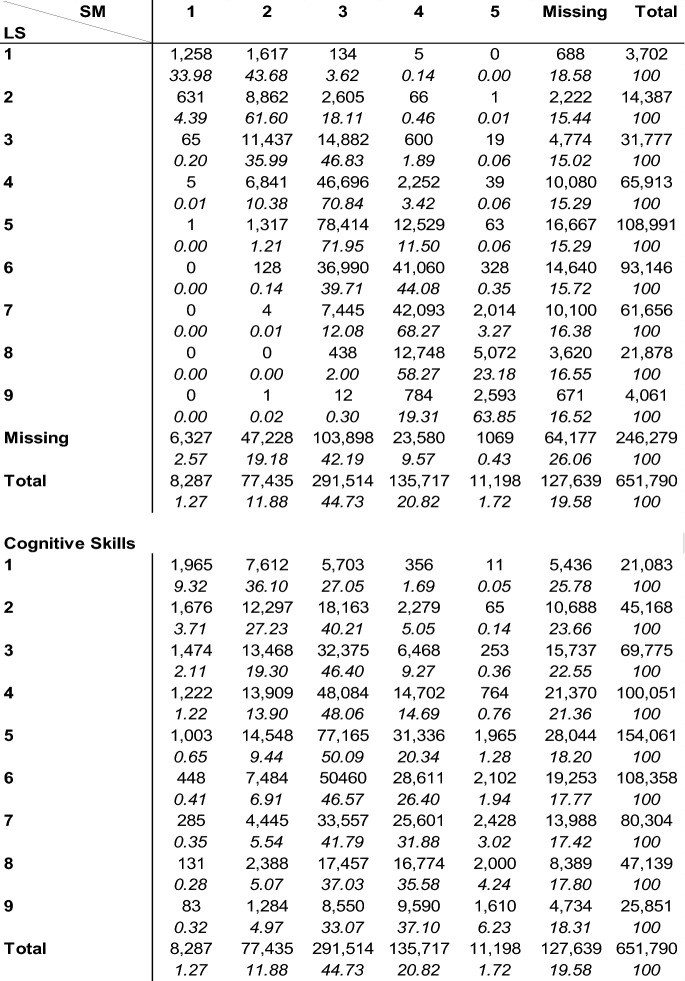
Descriptive statistics, frequency table with social maturity (SM) and leadership skills (LS)/cognitive skills, row percentages

**Table 16:**
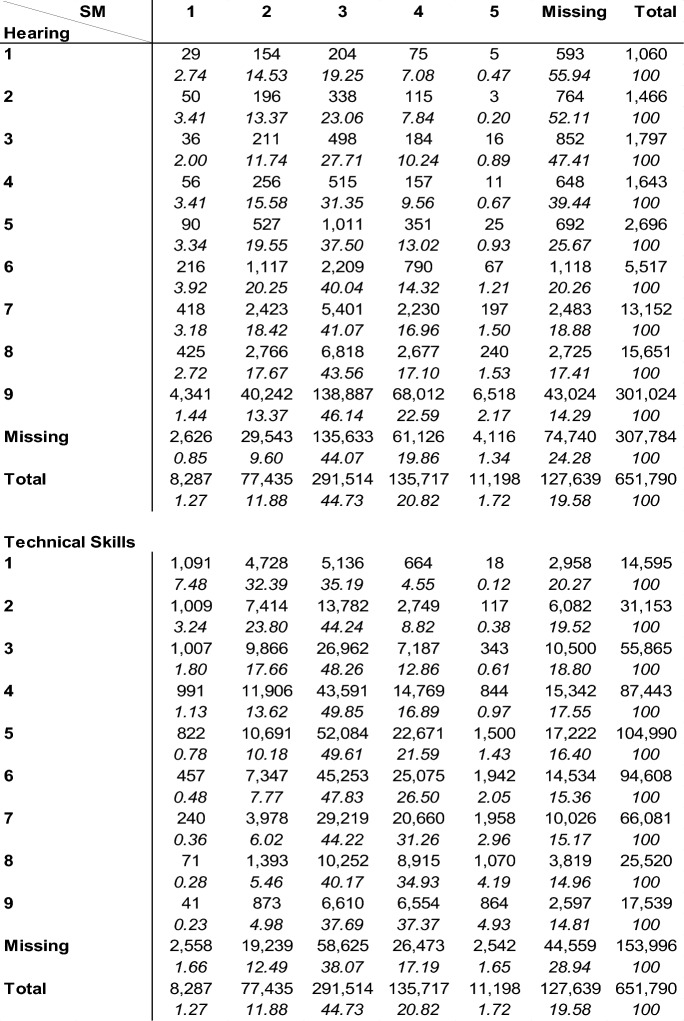
Descriptive statistics, frequency table with social maturity (SM) and hearing test scores/technical skills test scores, row percentages

## Appendix

### Additional Information on the Swedish Military Conscription

Some information has already been provided in the manuscript. Although several parts of the psychological interviews remain classified (e.g., the specific guidelines for the psychologists), some more details around the interviews and the military conscription have been made available by previous research (e.g., Lindqvist & Vestman, 2011; Nyberg et al., 2020).

For instance, Lindqvist and Vestman (2010, 2011) sketch the development of the psychological conscription interviews in the historical context and their relevance for the Swedish army (Tables 2, 3, 4, 5, 6, 7, 8, 9, 10, 11, 12, 13, 14, 15, 16). They report that psychological factors have been collected in the Swedish military since the early 1940’s when several tests (on IQ, physical health, psychological factors) were introduced for the entire cohort of recruits (Lindqvist & Vestman, 2010, 2011). The Swedish army has used prior experiences from Germany and the US to develop and establish the psychological assessment during military conscription (Lindqvist & Vestman, 2011). In the first years, psychological measures, by at that time advanced psychometric methods, should predict the recruits’ performances during military service (Lindqvist & Vestman, 2011), and indeed, the recruits’ skills were tested at later stages of the military service to examine the reliability of the psychological tests (Lindqvist & Vestman, 2010). Based on these first experiences, psychological interviews of around 10–20 min were introduced by 1950 in order to assess the psychological suitability of the young males for the military service (Lindqvist & Vestman, 2010). Regarding the birth cohorts that were used for this study (1963–1979), psychological interviews were already well-established, stemmed from 1970 and did not change before 1995 when only minor changes occurred (Lindqvist & Vestman, 2010, 2011; Ludvigsson et al., 2022).

All men of the analyzed sample (birth cohorts 1963–1979) had to attend the military conscription, typically when they were 18 or 19 years old (Lindqvist & Vestman, 2011), which lasted one or two days at the maximum (Mönstringshandboken, 2021). Using results from tests on several facets (e.g., IQ, physical health, psychological factors), one of the main goals of the psychological interviews was it to evaluate the recruit’s capacity to cope with mental stress that may appear during the military service (Carlstedt, 2000; Lindqvist & Vestman, 2011) without producing severe mental damages (Nyberg et al., 2020). Additionally, the psychologists may have detected psychological concerns regarding the mental health of the recruits leading to exclusion from military service (Nilsson et al., 2001). Reasons to exclude men from the military service were bad health, undemocratic attitudes, anti-social disorders, or obsession with the army (Ludvigsson et al., 2022). Low scores on cognitive (from IQ tests) or non-cognitive abilities (from psychological interviews) did not belong to the exclusion criteria but they were used to select males for certain positions within different fields of the military (Lindqvist & Vestman, 2011). Furthermore, first signs of poor mental health detected by the psychologist were further examined by conscription physicians who may have diagnosed males with mental diseases according to the ICD-code of the WHO (Nyberg et al., 2020).

### Psychological Measures

Psychologists were asked to rate several psychological factors such as social maturity, psychological energy, intensity, and emotional stability (Bihagen et al., 2013) on a scale from 1 to 5 (Lindqvist & Vestman, 2011). Perseverance, the capacity to maintain attention, and the ability to carry out goals are examples of psychological energy (Mood et al., 2012). Intensity is defined as the degree and regularity of one's leisure activities, as well as one's capacity to motivate oneself internally without assistance from others (Mood et al., 2012). Social maturity and emotional stability have received particularly high attention in previous research and are of particular high interest for the military, as shown in the sections below. The personality factor measures are generally based on questions about behaviors rather than attitudes (Bihagen et al., 2013; Mood et al., 2012).

The evaluation on the personality factors should serve as support for the assessment of the overall military aptitude (scores 1–9) (Lindqvist & Vestman, 2011; Mood et al., 2012). Higher scores on this scale stand for a better mental health and a higher military aptitude (Nilsson et al., 2001), and were assigned to men with relatively high levels of extraversion, independence, emotional stability, responsibility and persistence (Lindqvist & Vestman, 2011; Ludvigsson et al., 2022). This already indicates that two psychological fields were of particular interest for the Swedish military: (1) social skills, and (2) emotional stability and stress resilience. Therefore, psychological capacities and facets in relation to these factors were prominent in the psychological interviews. For instance, social relationships were subject of the interviews (Ministry of Defense Sweden, 1984) as well as the abilities to adapt to new circumstances and social surrounding (Lindqvist & Vestman, 2011; Ludvigsson et al., 2022). Moreover, certain emotional capacities were discussed including stress resilience (Nyberg et al., 2020) and fears such as claustrophobia or the fear of heights (Ludvigsson et al., 2022).

Apart from the personality factors described above, recruits were also assessed according to their leadership skills (LS). Although LS have been assessed independently of military aptitude and personality traits, it remains highly correlated with these other psychological factors (Mood et al., 2012). According to Ministry of Defense Sweden (1984), the leadership measure is based on a number of characteristics, including responsibility, mental acuity, and dominance (Figs. 6, 7, 8, 9, 10, 11, 12, 13, 14, 15, 16, 17, 18, 19, 20, 21, 22, 23, 24). During the interview, young men were also questioned about their personal capacity to mentor their friends (Lindqvist & Vestman, 2010). Men selected for officer positions were required to undergo leadership training, regardless of their desire to become an officer in the army, based on their LS scores from the conscription interview (Ministry of Defense Sweden, 1984).

But not every person has had their LS measured during the military conscription. According to Carlstedt (1998), the majority of males who scored on LS had previously completed cognitive tests during the conscription process and performed reasonably well (at least receiving a five on a scale of one to nine). This means that these men fall into the upper half of the IQ distribution (Carlstedt, 2000; Lindqvist & Vestman, 2010; Mårdberg & Carlstedt, 1998). As a result, in order to be considered for military officer posts (such as sergeants and lieutenants), applicants had to be somewhat clever and receive exceptionally high LS scores from qualified psychologists. For example, subsequent sergeants had to have received an IQ score of six or higher during the conscription, while lieutenants had to receive a score of seven or higher (Ludvigsson et al., 2022).

Psychologists were further required to produce a formal report on all measured psychological factors (Nilsson et al., 2001). Performing well during the military conscription, e.g., receiving high scores on social maturity, emotional stability, and LS from the psychological interviews, or high IQ scores from the cognitive tests, is positively linked with a number of later life outcomes. For instance, high scores on social maturity and emotional stability are positively linked with receiving higher income in later career outside the military (Bihagen et al., 2013). Furthermore, conscription-test-derived cognitive skills have been positively correlated with the likelihood of holding a managerial position following military service in the 30- to 40-year-old age range (Grönqvist & Lindqvist, 2015). Additionally, both having served as a higher officer in the military and having high cognitive skills evaluated during conscription are positively correlated with the likelihood of completing postsecondary education (Grönqvist & Lindqvist, 2015). As a result, military commanders may profit from their knowledge and expertise in the civilian workforce as well. This may have an impact on family formation processes by ensuring stable employment and making resources for family assistance available.

### The Role of the Recruits’ Psychological Constitution for the Swedish Military

The psychological evaluation of the recruits has been of particular interest for the Swedish army for many decades (Lindqvist & Vestman, 2010). For instance, stress resilience of the young men was an essential capability to evaluate for the military. High stress resilience has been characterized by high emotional stability, persistence, social skills, and independence, whereas opposite factors such as introversion, neuroticism, or adaptation difficulties have represented low stress resilience (Nyberg et al., 2020). Stress resilience has been considered as general indicator for general personality since it may be associated with a set of personality factors (e.g. emotional stability, extraversion, or conscientiousness), as argued by previous research (Falkstedt et al., 2013; Nyberg et al., 2020). This may be one reason for the essential role of stress resilience for military considerations.

Leadership skills (LS) and personality traits have been measured to choose suitable candidates for officer or other military roles (Lindqvist & Vestman, 2010; Ministry of Defense Sweden, 1984). The Swedish army may specifically target improving team performance in relation to officer candidates if team leaders exhibit qualities deemed appropriate for roles with greater responsibility (Grönqvist & Lindqvist, 2015). For example, prior studies have demonstrated that leaders who score higher on positive traits (such compassion and self-assurance) outperform their peers when it comes to team coordination (Chidester et al., 1991). Additionally, some evidence indicates a positive correlation between a team leader's social abilities and the team's effectiveness (Chidester et al., 1990).

### Five Topics in the Interviews

Psychological interviews should address five topics about experiences, behaviors and circumstances in life (Lindqvist & Vestman, 2011). By examining these topics, the military has aimed to get assessments on the recruits’ capability to adopt to new situations and environments (Nyberg et al., 2020). Accordingly, the young males were asked about behavioral experiences such as quitting school or work, or confrontations with supervisors (Lindqvist & Vestman, 2010; Nyberg et al., 2020). The interviews should provide the military with accurate judgements on the psychological constitution of the recruits, with particular focus on social skills, responsibility, independence, or other capabilities that may be beneficial for the military service and, in worst case, for war (Nyberg et al., 2020).

As first topic, the school experience was recorded, including academic successes as well as encounters with the school's social environment and individual experiences (Lindqvist & Vestman, 2010; Nyberg et al., 2020). The military, for example, sought to find out if the conscript had to repeat any classes, had to drop out of school, or had to stop altogether (Lindqvist & Vestman, 2010; Nyberg et al., 2020).

Second, in order to assess the recruits' capacity to adapt to the workplace, interviews should focus on the men's work experience (Lindqvist & Vestman, 2010; Nyberg et al., 2020). As with school experiences, behavioral aspects rather than views were of interest, such as conflicts with coworkers or supervisors, past firings, and past resignations from employment (Lindqvist & Vestman, 2010; Nyberg et al., 2020). Psychologists should assess the recruits based on their future career intentions (e.g., whether they exist, how feasible they are, etc.) if they had little to no job experience (Lindqvist & Vestman, 2010).

Third, the subjects of the interviews discussed their free time (Ministry of Defense Sweden, 1984; Nyberg et al., 2020). Specifically, the interviews focused on the recruits' fervent and active pursuit of interests and hobbies (Lindqvist & Vestman, 2010). The psychologist considers a number of aspects of these activities, such as whether they show more extraversion or introversion, if they participate in team sports or not, how diverse the activities are, and whether the recruits could fit in well in a particular situation (Lindqvist & Vestman, 2010; Nyberg et al., 2020). The question about recruits' perceptions of their own capacity to lead peers must be included in this field. This is a crucial indicator for psychologists assessing the male recruits' leadership abilities (Lindqvist & Vestman, 2010).

The fourth theme is on family history, including interactions with parents and siblings (Lindqvist & Vestman, 2010; Ministry of Defense Sweden, 1984). Here, the recruits' subjective reports and experiences about their living arrangements are particularly interesting because the objective is to assess how effectively the men can adjust to challenging circumstances and how much they rely on their parents (Lindqvist & Vestman, 2010; Nyberg et al., 2020). How frequently the recruit consumes alcohol is one essential question to ask in this part of the interview (Lindqvist & Vestman, 2010).

The final subject is the recruits' emotional stability (Lindqvist & Vestman, 2010; Nyberg et al., 2020). Additionally, the interviewer gets the chance to revisit remarks made earlier in the session or specific responses from test results from previous phases of the conscription process (Lindqvist & Vestman, 2010). The psychologist may consider the degree of maturity and the self-knowledge of the recruits (Lindqvist & Vestman, 2010; Nyberg et al., 2020).

Throughout the interview, the psychologists were asked to talk in a civilian and neutral manner, and avoid interrupting the recruits (Lindqvist & Vestman, 2010). The psychologist should also refrain from giving the impression that the interview’s purpose is only to screen the men according to their psychological capabilities but rather emphasize that the psychologist may provide mental support (Lindqvist & Vestman, 2010). Finally, the psychologists had access to information from prior steps of the conscription procedure such as the IQ test results (Ludvigsson et al., 2022; Nyberg et al., 2020), physical health, or replies to questionnaires on family, friends and hobbies (Lindqvist & Vestman, 2011; Ludvigsson et al., 2022). Moreover, psychologists had school grades, marital status and previous work experience of the recruits available (Lindqvist & Vestman, 2010; Nyberg et al., 2020).

### Advantages of Psychological Interviews Over Standardized Questionnaires

Psychological interviews have been a useful tool for the military to reach its goals of selecting suitable candidates for different positions in the army, and deselecting unsuitable ones (e.g., neurotics, psychopaths) (Ludvigsson et al., 2022). When filtering unsuitable men out, standardized pen-and-paper questionnaires have the disadvantage that intelligent (but unsuitable) persons may manipulate their answers relatively easily (Lindqvist & Vestman, 2011), i.e., they may simply tick answers that promise higher chances to get accepted. Qualified and trained psychologists have the chance to recognize males with severe mental disorders or undemocratic values during the personal interview (Ludvigsson et al., 2022). Similarly, the selection of suitable candidates for officer (Larsson & Kallenberg, 2006; Lindqvist & Vestman, 2010) or other military positions (Carlstedt, 1998, 2000) has been of high priority, and professional evaluations by experts may be more promising than self-reports to reach this goal.

Psychologists have been provided with certain guidelines on how to conduct the interviews (Nyberg et al., 2020) but specific details on these instructions have not been publicly available (Nilsson et al., 2001; Nyberg et al., 2020). Furthermore, the military did not provide the psychologists with specific question formulations so that the interviewers were free in how to phrase the questions (Nyberg et al., 2020).

Around 1–2% of the conscripts from each cohort was prohibited from military service due to psychological and medical concerns (Carlstedt, 1998, 2000) whereas approximately 10% did not end up in the actual military service (Lindqvist & Vestman, 2011), probably kept as training reserves (Carlstedt, 1998). This selection based on psychological assessments would not be similarly accurate using self-reports. Therefore, around 90% of the conscripted males had to attend the military service (Lindqvist & Vestman, 2011).

The relevance for choosing officers as wisely as possible and the necessity of looking for suitable candidates among the recruits may lie in the hierarchical structure of the Swedish military. Around 90% of the Swedish military officers are low-level officers who leave the military after approximately one year (Grönqvist & Lindqvist, 2015). Approximately one third of each birth cohort of conscripted men receive training for the officer positions in the lower hierarchy (Grönqvist & Lindqvist, 2015). For instance, approximately 10% of the recruited males were trained for 12–18 months as non-commissioned officer in order to lead groups of platoon (Lindqvist & Vestman, 2011) or company size (30–120 recruits) (Carlstedt, 1998). However, most males that get an officer position (around 23% of the total recruits) were trained for around 10 months in order to lead smaller groups (squads) (Lindqvist & Vestman, 2011) with typically eight to ten recruits (Carlstedt, 1998). The other recruits that are not selected as officers (around 67%) served in the army for seven or eight months (Lindqvist & Vestman, 2011). Recruits typically started the army service one or two years after the conscription in assigned branches of the army according to psychological and physical abilities (Carlstedt, 1998).

## References

[CR1] Ajzen, I., & Klobas, J. (2013). Fertility intentions: An approach based on the theory of planned behavior. *Demographic Research,**29*, 203–232. 10.4054/DemRes.2013.29.8

[CR2] Aldén, L., Boschini, A. D., & Sundström, M. (2022). Who becomes a father? The rising importance of non-cognitive ability. *SSRN Electronic Journal*. 10.2139/ssrn.4207533

[CR3] Allemand, M., Hill, P. L., & Lehmann, R. (2015). Divorce and personality development across middle adulthood: Divorce and personality development. *Personal Relationships,**22*(1), 122–137. 10.1111/pere.12067

[CR4] Allen, M. S. (2019). The role of personality in sexual and reproductive health. *Current Directions in Psychological Science,**28*(6), 581–586. 10.1177/0963721419862293

[CR150] Allen, M. S., & Desille, A. E. (2017). Personality and sexuality in older adults. *Psychology and Health, 32*(7), 843–859. 10.1080/08870446.2017.1307373 28335615

[CR5] Alvergne, A., Jokela, M., & Lummaa, V. (2010). Personality and reproductive success in a high-fertility human population. *Proceedings of the National Academy of Sciences of the United States of America,**107*(26), 11745–11750. 10.1073/pnas.1001752107 20538974 10.1073/pnas.1001752107 PMC2900694

[CR6] Andersson, G., & Scott, K. (2007). Childbearing dynamics of couples in a universalistic welfare state: The role of labor-market status, country of origin, and gender. *Demographic Research,**17*, 897–938. 10.4054/DemRes.2007.17.30

[CR7] Angelov, N., Johansson, P., & Lindahl, E. (2016). Parenthood and the gender gap in pay. *Journal of Labor Economics,**34*(3), 545–579. 10.1086/684851

[CR8] Angrist, J. D., & Pischke, J.-S. (2009). *Mostly harmless econometrics: An empiricist’s companion*. Princeton University Press.

[CR9] Asendorpf, J. B., & Wilpers, S. (1998). Personality effects on social relationships. *Journal of Personality and Social Psychology,**74*(6), 1531–1544. 10.1037/0022-3514.74.6.1531

[CR10] Ashton, M. C., & Lee, K. (2005). Honesty-humility, the Big Five, and the Five-Factor model. *Journal of Personality,**73*(5), 1321–1354. 10.1111/j.1467-6494.2005.00351.x 16138875 10.1111/j.1467-6494.2005.00351.x

[CR11] Avison, M., & Furnham, A. (2015). Personality and voluntary childlessness. *Journal of Population Research,**32*(1), 45–67. 10.1007/s12546-014-9140-6

[CR12] Barclay, K., & Kolk, M. (2020). The influence of health in early adulthood on male fertility. *Population and Development Review,**46*(4), 757–785. 10.1111/padr.12357

[CR13] Bihagen, E., Nermo, M., & Stern, C. (2013). Class origin and elite position of men in business firms in Sweden, 1993–2007: The importance of education, cognitive ability, and personality. *European Sociological Review,**29*(5), 939–954. 10.1093/esr/jcs070

[CR14] Bleidorn, W., Hopwood, C. J., & Lucas, R. E. (2018). Life events and personality trait change: Life events and trait change. *Journal of Personality,**86*(1), 83–96. 10.1111/jopy.12286 27716921 10.1111/jopy.12286

[CR15] Boertien, D., & Mortelmans, D. (2018). Does the relationship between personality and divorce change over time? A cross-country comparison of marriage cohorts. *Acta Sociologica,**61*(3), 300–316. 10.1177/0001699317709048

[CR16] Boertien, D., von Scheve, C., & Park, M. (2017). Can personality explain the educational gradient in divorce? Evidence from a nationally representative panel survey. *Journal of Family Issues,**38*(10), 1339–1362. 10.1177/0192513X15585811

[CR17] Botwin, M. D., Buss, D. M., & Shackelford, T. K. (1997). Personality and mate preferences: Five factors in mate selection and marital satisfaction. *Journal of Personality,**65*(1), 107–136. 10.1111/j.1467-6494.1997.tb00531.x 9143146 10.1111/j.1467-6494.1997.tb00531.x

[CR18] Boyce, C. J., Wood, A. M., & Ferguson, E. (2016). For better or for worse: The moderating effects of personality on the marriage-life satisfaction link. *Personality and Individual Differences,**97*, 61–66. 10.1016/j.paid.2016.03.005

[CR19] Bumpass, L. L., Castro Martin, T., & Sweet, J. A. (1991). The impact of family background and early marital factors on marital disruption. *Journal of Family Issues,**12*(1), 22–42. 12316638 10.1177/019251391012001003

[CR20] Buss, D. M. (1994). The strategies of human mating. *American Scientist,**82*(3), 238–249.

[CR21] Buss, D. M. (2006). Strategies of human mating. *Psychological Topics,**15*(2), 239–260.

[CR22] Buss, D. M., & Barnes, M. (1986). Preferences in human mate selection. *Journal of Personality and Social Psychology,**50*(3), Article Article 3.

[CR23] Buyukkececi, Z., & Leopold, T. (2021). Sibling influence on family formation: A study of social interaction effects on fertility, marriage, and divorce. *Advances in Life Course Research,**47*, Article 100359. 10.1016/j.alcr.2020.100359 36715429 10.1016/j.alcr.2020.100359

[CR24] Bygren, M., Gähler, M., & Magnusson, C. (2021). The constant gap: Parenthood premiums in Sweden 1968–2010. *Social Forces,**100*(1), 137–168. 10.1093/sf/soaa097

[CR25] Caldwell, D. F., & Burger, J. M. (1998). Personality charateristics of job applicants and success in screening interviews. *Personnel Psychology,**51*(1), 119–136. 10.1111/j.1744-6570.1998.tb00718.x

[CR26] Carlstedt, B. (2000). *Cognitive abilities—Aspects of structure, process and measurement*. University of Gothenburg.

[CR27] Carlstedt, B. (1998). Validation of psychological predictors in the Swedish enlistment system. *Navy Advancement Center*. 10.1037/e623442007-001

[CR28] Chamorro-Premuzic, T., & Furnham, A. (2003). Personality traits and academic examination performance. *European Journal of Personality,**17*(3), 237–250. 10.1002/per.473

[CR29] Chidester, R., Kanki, G., Research, A., Clayton, H., Dickinson, L., Park, M., & Bowles, V. (1990). Personality factors in flight operations: Volume I. Leader characteristics and crew performance in a full-mission air transport simulation. *NASA Technical Memorandum*, *102259*.

[CR30] Chidester, T. R., Helmreich, R. L., Gregorich, S. E., & Geis, C. E. (1991). Pilot personality and crew coordination: Implications for training and selection. *The International Journal of Aviation Psychology,**1*(1), 25–44. 10.1207/s15327108ijap0101_3 11539104 10.1207/s15327108ijap0101_3

[CR31] Cook, K. W., Vance, C. A., & Spector, P. E. (2000). The relation of candidate personality with selection-interview outcomes. *Journal of Applied Social Psychology,**30*(4), 867–885. 10.1111/j.1559-1816.2000.tb02828.x

[CR32] Cools, S., & Hart, R. K. (2017). The effect of childhood family size on fertility in adulthood: New evidence from IV estimation. *Demography,**54*(1), 23–44. 10.1007/s13524-016-0537-z 28032264 10.1007/s13524-016-0537-z

[CR33] Costa, P. T., Jr., Herbst, J. H., & McCrae, R. R. (2000). Personality at midlife: Stability, intrinsic maturation, and response to life events. *Assessment,**7*(4), 365–378. 11151962 10.1177/107319110000700405

[CR34] Cummins, D. (2006). Dominance, status, and social hierarchies. In D. M. Buss (Ed.), *The handbook of evolutionary psychology* (pp. 676–697). Wiley. 10.1002/9780470939376.ch23

[CR35] Dahlberg, J., & Kolk, M. (2018). Explaining Swedish sibling similarity in fertility: Parental fertility behavior vs. social background. *Demographic Research,**39*, 883–896. 10.4054/DemRes.2018.39.32

[CR36] Damian, R. I., Su, R., Shanahan, M., Trautwein, U., & Roberts, B. W. (2015). Can personality traits and intelligence compensate for background disadvantage? Predicting status attainment in adulthood. *Journal of Personality and Social Psychology,**109*(3), 473–489. 10.1037/pspp0000024 25402679 10.1037/pspp0000024 PMC4433862

[CR37] Donnellan, M. B., Conger, R. D., & Bryant, C. M. (2004). The big five and enduring marriages. *Journal of Research in Personality,**38*(5), 481–504. 10.1016/j.jrp.2004.01.001

[CR38] Dronkers, J., & Hox, J. (2006). The importance of the common family background for the similarity of divorce risks of siblings: A multi-level event history analysis. In *Research in multi level issues* (Vol. 5, pp. 217–238). Emerald (MCB UP ). 10.1016/S1475-9144(06)05010-7

[CR39] Dupuy, A., & Galichon, A. (2014). Personality traits and the marriage market. *Journal of Political Economy,**122*(6), 1271–1319.

[CR40] Duvander, A.-Z., & Kridahl, L. (2020). Decisions on marriage? Couples’ decisions on union transition in Sweden. *Genus,**76*(1), 1–21. 10.1186/s41118-020-00092-5

[CR41] Epifanio, M. S., Genna, V., De Luca, C., Roccella, M., & La Grutta, S. (2015). Paternal and maternal transition to parenthood: The risk of postpartum depression and parenting stress. *Pediatric Reports*. 10.4081/pr.2015.5872 10.4081/pr.2015.5872 PMC4508624 26266033

[CR42] Eurostat. (2018). *Marriage now more common in Sweden*. https://ec.europa.eu/eurostat/statistics-explained/index.php?title=Archive:Marriages_and_births_in_Sweden&oldid=396647#More_children_per_woman_during_the_2000s.

[CR43] Falkstedt, D., Sorjonen, K., Hemmingsson, T., Deary, I. J., & Melin, B. (2013). Psychosocial functioning and intelligence both partly explain socioeconomic inequalities in premature death. A population-based male cohort study. *PLoS ONE,**8*(12), Article e82031. 10.1371/journal.pone.0082031 24349174 10.1371/journal.pone.0082031 PMC3859588

[CR44] Fieder, M., Huber, S., Bookstein, F. L., Iber, K., Schafer, K., Winckler, G., & Wallner, B. (2005). Status and reproduction in humans: New evidence for the validity of evolutionary explanations on basis of a university sample. *Ethology,**111*(10), 940–950. 10.1111/j.1439-0310.2005.01129.x

[CR45] Figueredo, A. J., Sefcek, J. A., & Jones, D. N. (2006). The ideal romantic partner personality. *Personality and Individual Differences,**41*(3), 431–441. 10.1016/j.paid.2006.02.004

[CR46] Fisher, T. D., & McNulty, J. K. (2008). Neuroticism and marital satisfaction: The mediating role played by the sexual relationship. *Journal of Family Psychology,**22*(1), 112–122. 10.1037/0893-3200.22.1.112 18266538 10.1037/0893-3200.22.1.112

[CR47] Frejka, T. (2008). Overview chapter 3: Birth regulation in Europe: Completing the contraceptive revolution. *Demographic Research,**19*, 73–84. 10.4054/DemRes.2008.19.5

[CR48] Frejka, T., Sobotka, T., Hoem, J. M., & Toulemon, L. (2008). Summary and general conclusions: Childbearing trends and policies in Europe. *Demographic Research,**19*, 5–14. 10.4054/DemRes.2008.19.2

[CR49] Friedman, D., Hechter, M., & Kanazawa, S. (1994). A theory of the value of children. *Demography,**31*(3), 375–401. 7828763

[CR50] Gershuny, B. S., & Sher, K. J. (1998). The relation between personality and anxiety: Findings from a 3-year prospective study. *Journal of Abnormal Psychology,**107*(2), 252–262. 9604554 10.1037//0021-843x.107.2.252

[CR51] Goldberg, L. R. (1993). The structure of phenotypic personality traits. *American Psychologist,**48*(1), 26–34. 8427480 10.1037//0003-066x.48.1.26

[CR52] Grönqvist, E., & Lindqvist, E. (2015). *Kan man lära sig ledarskap? Befälsutbildning under värnplikten och utfall på arbetsmarknaden* (4). Institutet för arbetsmaknads- och utbildningspolitisk utvärdering.

[CR53] Harris, M. A., Brett, C. E., Johnson, W., & Deary, I. J. (2016). Personality stability from age 14 to age 77 years. *Psychology and Aging,**31*(8), 862–874. 10.1037/pag0000133 27929341 10.1037/pag0000133 PMC5144810

[CR54] Hofmann, E., & Krapf, S. (2024). “Like two peas in a pod?” Homogamous personalities, education, and union dissolution. *Genus,**80*(1), 19. 10.1186/s41118-024-00229-w

[CR55] Holland, A. S., & Roisman, G. I. (2008). Big five personality traits and relationship quality: Self-reported, observational, and physiological evidence. *Journal of Social and Personal Relationships,**25*(5), 811–829. 10.1177/0265407508096697

[CR56] Hopcroft, R. L. (2006). Sex, status, and reproductive success in the contemporary United States. *Evolution and Human Behavior,**27*(2), 104–120. 10.1016/j.evolhumbehav.2005.07.004

[CR57] Hopwood, C. J., & Bleidorn, W. (2018). Stability and change in personality and personality disorders. *Current Opinion in Psychology,**21*, 6–10. 10.1016/j.copsyc.2017.08.034 28923391 10.1016/j.copsyc.2017.08.034

[CR58] Hutteman, R., Bleidorn, W., Penke, L., & Denissen, J. J. A. (2013). It takes two: A longitudinal dyadic study on predictors of fertility outcomes: Dyadic predictors of fertility outcomes. *Journal of Personality,**81*(5), 487–498. 10.1111/jopy.12006 22925070 10.1111/jopy.12006

[CR59] Jalovaara, M. (2001). Socio-economic status and divorce in first marriages in Finland 1991–93. *Population Studies,**55*(2), 119–133. 10.1080/00324720127685

[CR60] Jalovaara, M. (2003). The joint effects of marriage partners’ socioeconomic positions on the risk of divorce. *Demography,**40*(1), 67–81. 12647514 10.1353/dem.2003.0004

[CR61] Jalovaara, M. (2013). Socioeconomic resources and the dissolution of cohabitations and marriages. *European Journal of Population = Revue Européenne De Démographie,**29*(2), 167–193. 10.1007/s10680-012-9280-3

[CR62] Jalovaara, M., Neyer, G., Andersson, G., Dahlberg, J., Dommermuth, L., Fallesen, P., & Lappegård, T. (2019). Education, gender, and cohort fertility in the Nordic countries. *European Journal of Population,**35*(3), 563–586. 10.1007/s10680-018-9492-2 31372105 10.1007/s10680-018-9492-2 PMC6639448

[CR63] Jockin, V., McGue, M., & Lykken, D. T. (1996). Personality and divorce: A genetic analysis. *Journal of Personality and Social Psychology,**71*(2), 288–299. 8765483 10.1037//0022-3514.71.2.288

[CR64] Johns, S. E., Dickins, T. E., & Clegg, H. T. (2011). Teenage pregnancy and motherhood: How might evolutionary theory inform policy? *Journal of Evolutionary Psychology,**9*(1), 3–19. 10.1556/JEP.9.2011.37.122947949

[CR65] Johnson, A. B., & Rodgers, J. L. (2006). The impact of having children on the lives of women: The effects of children questionnaire. *Journal of Applied Social Psychology,**36*(11), 2685–2714. 10.1111/j.0021-9029.2006.00123.x

[CR66] Johnson, W., McGue, M., Krueger, R. F., & Bouchard, T. J. (2004). Marriage and personality: A genetic analysis. *Journal of Personality and Social Psychology,**86*(2), 285–294. 10.1037/0022-3514.86.2.285 14769084 10.1037/0022-3514.86.2.285

[CR67] Jokela, M. (2012). Birth–cohort effects in the association between personality and fertility. *Psychological Science,**23*(8), 835–841. 10.1177/0956797612439067 22722269 10.1177/0956797612439067

[CR68] Jokela, M., Alvergne, A., Pollet, T. V., & Lummaa, V. (2011). Reproductive behavior and personality traits of the five factor model. *European Journal of Personality,**25*(6), 487–500. 10.1002/per.822

[CR69] Jokela, M., Kivimäki, M., Elovainio, M., & Keltikangas-Järvinen, L. (2009). Personality and having children: A two-way relationship. *Journal of Personality and Social Psychology,**96*(1), 218–230. 10.1037/a0014058 19210076 10.1037/a0014058

[CR70] Jokela, M., Pekkarinen, T., Sarvimäki, M., Terviö, M., & Uusitalo, R. (2017). Secular rise in economically valuable personality traits. *Proceedings of the National Academy of Sciences of the United States of America,**114*(25), 6527–6532. 10.1073/pnas.1609994114 28584092 10.1073/pnas.1609994114 PMC5488912

[CR71] Jonason, P. K., Koehn, M. A., Okan, C., & O’Connor, P. J. (2018). The role of personality in individual differences in yearly earnings. *Personality and Individual Differences,**121*, 170–172. 10.1016/j.paid.2017.09.038

[CR72] Judge, T. A., Higgins, C. A., Thoresen, C. J., & Barrick, M. R. (1999). The big five personality traits, general mental ability, and career success across the life span. *Personnel Psychology,**52*(3), 621–652. 10.1111/j.1744-6570.1999.tb00174.x

[CR73] Judge, T. A., & Ilies, R. (2002). Relationship of personality to performance motivation: A meta-analytic review. *Journal of Applied Psychology,**87*(4), 797–807. 10.1037/0021-9010.87.4.797 12184582 10.1037/0021-9010.87.4.797

[CR74] Kandler, C., & Bleidorn, W. (2015). Personality Differences and development: Genetic and environmental contributions. In *International Encyclopedia of the Social & Behavioral Sciences* (pp. 884–890). Elsevier. 10.1016/B978-0-08-097086-8.25011-3

[CR75] Kandler, C. (2012). Nature and nurture in personality development: The case of neuroticism and extraversion. *Current Directions in Psychological Science,**21*(5), 290–296.

[CR76] Karney, B. R., & Bradbury, T. N. (1997). Neuroticism, marital interaction, and the trajectory of marital satisfaction. *Journal of Personality and Social Psychology,**72*(5), 1075–1092. 9150586 10.1037//0022-3514.72.5.1075

[CR77] Knoester, C., & Booth, A. (2000). Barriers to divorce. When are they effective? When are they not? *Journal of Family Issues,**21*(1), 78–99.

[CR78] Kolk, M. (2015). The causal effect of an additional sibling on completed fertility: An estimation of intergenerational fertility correlations by looking at siblings of twins. *Demographic Research,**32*, 1409–1420. 10.4054/DemRes.2015.32.51

[CR79] Kolk, M. (2019). The relationship between lifecourse accumulated income and childbearing of Swedish men and women born 1940–1970. *Stockholm Research Reports in Demography,**19*, 1–31. 10.1080/00324728.2022.2134578

[CR80] Kolk, M., & Barclay, K. (2019). Cognitive ability and fertility among Swedish men born 1951–1967: Evidence from military conscription registers. *Proceedings of the Royal Society b: Biological Sciences,**286*(1902), 20190359. 10.1098/rspb.2019.0359 10.1098/rspb.2019.0359 PMC6532519 31064299

[CR81] Kolk, M., & Barclay, K. (2021). Do income and marriage mediate the relationship between cognitive ability and fertility? Data from Swedish taxation and conscriptions registers for men born 1951–1967. *Intelligence,**84*, 1–11. 10.1016/j.intell.2020.101514

[CR82] Kramarz, F., Rosenqvist, O., & Skans, O. N. (2021). How family background shapes the relationship between human capital and fertility. *Journal of Population Economics*. 10.1007/s00148-021-00834-5

[CR83] Larsson, G., & Kallenberg, K. (2006). *Direkt ledarskap*. Förvarsmakten.

[CR84] Lavner, J. A., Weiss, B., Miller, J. D., & Karney, B. R. (2018). Personality change among newlyweds: Patterns, predictors, and associations with marital satisfaction over time. *Developmental Psychology,**54*(6), 1172–1185. 10.1037/dev0000491 29251970 10.1037/dev0000491 PMC5962362

[CR85] Le, B., Dove, N. L., Agnew, C. R., Korn, M. S., & Mutso, A. A. (2010). Predicting nonmarital romantic relationship dissolution: A meta-analytic synthesis. *Personal Relationships,**17*(3), Article Article 3. 10.1111/j.1475-6811.2010.01285.x

[CR86] Leikas, S., & Salmela-Aro, K. (2015). Personality trait changes among young Finns: The role of life events and transitions: Personality change and life events. *Journal of Personality,**83*(1), 117–126. 10.1111/jopy.12088 24444435 10.1111/jopy.12088

[CR87] Leone, C., & Hawkins, B. L. (2019). Cohabitation vs. marriage: Self-monitoring and self-selection to intimate relationships. *Journal of Psychology & Behavioral Science*. 10.15640/jpbs.v7n2a2

[CR88] Lesthaeghe, R. (2014). The second demographic transition: A concise overview of its development. *Proceedings of the National Academy of Sciences,**11*(51), 18112–18115. 10.1073/pnas.1420441111 PMC4280616 25453112

[CR89] Li, N. P., Bailey, J. M., Kenrick, D. T., & Linsenmeier, J. A. W. (2002). The necessities and luxuries of mate preferences: Testing the tradeoffs. *Journal of Personality and Social Psychology,**82*(6), Article Article 6. 10.1037/0022-3514.82.6.947 12051582

[CR90] Lindqvist, E., & Vestman, R. (2010). *Web appendix (B-F): The labor market returns to cognitive and noncognitive ability: Evidence from the Swedish enlistment*. https://assets.aeaweb.org/asset-server/articles-attachments/aej/app/app/2009-0140_app.pdf

[CR91] Lindqvist, E., & Vestman, R. (2011). The labor market returns to cognitive and noncognitive ability: Evidence from the Swedish enlistment. *American Economic Journal: Applied Economics,**3*(1), 101–128. 10.1257/app.3.1.101

[CR92] Lucas, R. E., & Donnellan, M. B. (2011). Personality development across the life span: Longitudinal analyses with a national sample from Germany. *Journal of Personality and Social Psychology,**101*(4), 847–861. 10.1037/a0024298 21707197 10.1037/a0024298

[CR93] Ludvigsson, J. F., Berglind, D., Sundquist, K., Sundström, J., Tynelius, P., & Neovius, M. (2022). The Swedish military conscription register: Opportunities for its use in medical research. *European Journal of Epidemiology*. 10.1007/s10654-022-00887-0 10.1007/s10654-022-00887-0 PMC9329412 35810240

[CR94] Lundberg, S. (2012). Personality and marital surplus. *IZA Journal of Labor Economics,**1*(1), 1–21. 10.1186/2193-8997-1-4 PMC4798751 26998417

[CR95] Lyngstad, T. H. (2004). The impact of parent’s and spouses’ education on divorce rates in Norway. *Demographic Research,**10*, 121–142. 10.4054/DemRes.2004.10.5

[CR96] Lyngstad, T. H., & Prskawetz, A. (2010). Do siblings’ fertility decisions influence each other? *Demography,**47*(4), 923–934. 10.1007/BF03213733 21308564 10.1007/BF03213733 PMC3000038

[CR97] Malouff, J. M., Thorsteinsson, E. B., Schutte, N. S., Bhullar, N., & Rooke, S. E. (2010). The five-factor model of personality and relationship satisfaction of intimate partners: A meta-analysis. *Journal of Research in Personality,**44*(1), 124–127. 10.1016/j.jrp.2009.09.004

[CR98] Mårdberg, B., & Carlstedt, B. (1998). Swedish enlistment battery (SEB): Construct validity and latent variable estimation of cognitive abilities by the CAT-SEB. *International Journal of Selection and Assessment,**6*(2), 107–114. 10.1111/1468-2389.00079

[CR99] McCrae, R. R., & Costa, P. T. (1987). Validation of the five-factor model of personality across instruments and observers. *Journal of Personality and Social Psychology,**52*(1), 81–90. 3820081 10.1037//0022-3514.52.1.81

[CR100] McGue, M., & Lykken, D. T. (1992). Genetic influence on risk of divorce. *Psychological Science,**3*(6), 368–373. 10.1111/j.1467-9280.1992.tb00049.x

[CR101] McLanahan, S., & Adams, J. (1987). Parenthood and psychological well-being. *Annual Review of Sociology,**13*(1), 237–257.

[CR102] McNulty, J. K. (2008). Neuroticism and interpersonal negativity: The independent contributions of perceptions and behaviors. *Personality and Social Psychology Bulletin,**34*(11), 1439–1450. 10.1177/0146167208322558 18703488 10.1177/0146167208322558

[CR103] Mencarini, L., Piccarreta, R., & Le Moglie, M. (2022). Life-course perspective on personality traits and fertility with sequence analysis. *Journal of the Royal Statistical Society: Series A (Statistics in Society),**185*(3), 1344–1369. 10.1111/rssa.12832

[CR104] Meyer, J., Fleckenstein, J., Retelsdorf, J., & Köller, O. (2019). The relationship of personality traits and different measures of domain-specific achievement in upper secondary education. *Learning and Individual Differences,**69*, 45–59. 10.1016/j.lindif.2018.11.005

[CR152] Miller, J. D., Lynam, D., Zimmerman, R. S., Logan, T. K., Leukefeld, C., & Clayton, R. (2004). The utility of the Five Factor Model in understanding risky sexual behavior. *Personality and Individual Differences, 36*(7), 1611–1626. 10.1016/j.paid.2003.06.009

[CR105] Miller, W. B. (1992). Personality traits and developmental experiences as antecedents of childbearing motivation. *Demography,**29*(2), 265–285. 10.2307/2061731 1607052

[CR106] Miller, W. B. (2011). Differences between fertility desires and intentions: Implications for theory, research and policy. *Vienna Yearbook of Population Research,**9*, 75–98.

[CR107] Ministry of Defense Sweden. (1984). *Conscription in the future* (71; Swedish Government Official Reports). https://lagen.nu/sou/1984:71?attachment=index.pdf&repo=soukb&dir=downloaded

[CR108] *Mönstringshandboken*. (2021). Plikt-och prövningsverket. https://pliktverket.se/download/18.3b29fb261791cde3ba045b1/1620048296684/Monstringshandboken-2021.pdf

[CR109] Mood, C., Jonsson, J. O., & Bihagen, E. (2012). Socioeconomic persistence across generations: Cognitive and noncognitive processes. In J. Ermisch, M. Jäntti, & T. Smeeding (Eds.), *From parents to children. The intergenerational transmission of advantage* (pp. 53–84). Russell Sage.

[CR110] Mood, C. (2010). Logistic regression: Why we cannot do what we think we can do, and what we can do about it. *European Sociological Review,**26*(1), 67–82. 10.1093/esr/jcp006

[CR111] Morosow, K., & Kolk, M. (2020). How does birth order and number of siblings affect fertility? A within-family comparison using Swedish register data. *European Journal of Population,**36*(2), 197–233. 10.1007/s10680-019-09525-0 32256257 10.1007/s10680-019-09525-0 PMC7113329

[CR153] Nettle, D. (2005). An evolutionary approach to the extraversion continuum. *Evolution and Human Behavior, 26*(4), 363–373. 10.1016/j.evolhumbehav.2004.12.004

[CR154] Nettle, D. (2006). The evolution of personality variation in humans and other animals. *American Psychologist, 61*(6), 622–631. 10.1037/0003-066X.61.6.622 10.1037/0003-066X.61.6.622 16953749

[CR112] Neyer, F. J., & Asendorpf, J. B. (2001). Personality–relationship transaction in young adulthood. *Journal of Personality and Social Psychology,**81*(6), 1190–1204. 10.1037/0022-3514.81.6.1190 11761317

[CR113] Nilsson, P. M., Nyberg, P., & Östergren, P.-O. (2001). Increased susceptibility to stress at a psychological assessment of stress tolerance is associated with impaired fetal growth. *International Journal of Epidemiology,**30*(1), 75–80. 10.1093/ije/30.1.75 11171861 10.1093/ije/30.1.75

[CR114] Nisén, J., Martikainen, P., Silventoinen, K., & Myrskylä, M. (2014). Age-specific fertility by educational level in the Finnish male cohort born 1940–1950. *Demographic Research,**31*, 119–136. 10.4054/DemRes.2014.31.5

[CR115] Noftle, E. E., & Shaver, P. R. (2006). Attachment dimensions and the big five personality traits: Associations and comparative ability to predict relationship quality. *Journal of Research in Personality,**40*(2), 179–208. 10.1016/j.jrp.2004.11.003

[CR116] Nyberg, J., Gustavsson, S., Åberg, M. A. I., Kuhn, H. G., & Waern, M. (2020). Late-adolescent risk factors for suicide and self-harm in middle-aged men: Explorative prospective population-based study. *The British Journal of Psychiatry,**217*(1), 370–376. 10.1192/bjp.2019.243 31690353 10.1192/bjp.2019.243

[CR117] Orth, U. (2013). How large are actor and partner effects of personality on relationship satisfaction? The importance of controlling for shared method variance. *Personality and Social Psychology Bulletin,**39*(10), 1359–1372. 23798373 10.1177/0146167213492429

[CR118] Orzeck, T., & Lung, E. (2005). Big-five personality differences of cheaters and non-cheaters. *Current Psychology,**24*(4), 274–286. 10.1007/s12144-005-1028-3

[CR119] Penke, L., Denissen, J. J. A., & Miller, G. F. (2007). The evolutionary genetics of personality. *European Journal of Personality,**21*(5), 549–587. 10.1002/per.629

[CR120] Penke, L., & Jokela, M. (2016). The evolutionary genetics of personality revisited. *Current Opinion in Psychology,**7*, 104–109. 10.1016/j.copsyc.2015.08.021

[CR121] Peters, S. (2023). The prospective power of personality for childbearing: A longitudinal study based on data from Germany. *Genus,**79*(1), 6. 10.1186/s41118-023-00184-y

[CR122] Pinquart, M., Stotzka, M., & Silbereisen, R. K. (2008). Personality and ambivalence in decisions about becoming parents. *Social Behavior and Personality,**36*(1), 87–96.

[CR123] Roberts, B. W., Chernyshenko, O. S., Stark, S., & Goldberg, L. R. (2005). The structure of conscientiousness: An empirical investigation based on seven major personality questionnaires. *Personnel Psychology,**58*(1), 103–139. 10.1111/j.1744-6570.2005.00301.x

[CR124] Roberts, B. W., Kuncel, N. R., Shiner, R., Caspi, A., & Goldberg, L. R. (2007). The power of personality: The comparative validity of personality traits, socioeconomic status, and cognitive ability for predicting important life outcomes. *Perspectives on Psychological Science,**2*(4), 313–345. 10.1111/j.1745-6916.2007.00047.x 26151971 10.1111/j.1745-6916.2007.00047.x PMC4499872

[CR125] Roelfs, D. J., Shor, E., Kalish, R., & Yogev, T. (2011). The rising relative risk of mortality for singles: Meta-analysis and meta-regression. *American Journal of Epidemiology,**174*(4), 379–389. 10.1093/aje/kwr111 21715646 10.1093/aje/kwr111

[CR126] Sandström, G., & Stanfors, M. (2020). Growing more equal and growing apart? Socioeconomic status and the rise of divorce in Sweden. *Lund Papers in Economic Demography,**4*, 1–45.

[CR155] Schmitt, D. P. (2004). The Big Five related to risky sexual behaviour across 10 world regions: Differential personality associations of sexual promiscuity and relationship infidelity. *European Journal of Personality, 18*(4), 301–319. 10.1002/per.520

[CR127] Schmitt, D. P., & Shackelford, T. K. (2008). Big Five Traits Related to Short-Term Mating: From Personality to Promiscuity across 46 Nations. *Evolutionary Psychology,**6*(2), 246–282. 10.1177/147470490800600204

[CR128] Senia, J. M., Neppl, T. K., Gudmunson, C. G., Donnellan, M. B., & Lorenz, F. O. (2016). The intergenerational continuity of socioeconomic status: Effects of parenting, personality, and age at first romantic partnership. *Journal of Family Psychology,**30*(6), 647–656. 10.1037/fam0000171 26651350 10.1037/fam0000171 PMC4907882

[CR129] Skirbekk, V., & Blekesaune, M. (2014). Personality traits increasingly important for male fertility: Evidence from Norway: Personality traits and male fertility. *European Journal of Personality,**28*, 521–529. 10.1002/per.1936

[CR130] Skirbekk, V., Blekesaune, M., & Sunde, H. F. (2025). Personality and fertility dynamics in Norway across four decades—Findings from the HUNT survey. *Personality and Individual Differences,**233*, Article 112862. 10.1016/j.paid.2024.112862

[CR131] Sobotka, T. (2008). Overview chapter 6: The diverse faces of the Second Demographic Transition in Europe. *Demographic Research,**19*, 171–224. 10.4054/DemRes.2008.19.8

[CR132] Sobotka, T., & Toulemon, L. (2008). Overview chapter 4: Changing family and partnership behaviour: Common trends and persistent diversity across Europe. *Demographic Research,**19*, 85–138. 10.4054/DemRes.2008.19.6

[CR133] Solomon, B. C., & Jackson, J. J. (2014). Why do personality traits predict divorce? Multiple pathways through satisfaction. *Journal of Personality and Social Psychology,**106*(6), 978–996. 10.1037/a0036190 24841100 10.1037/a0036190

[CR134] Spikic, S., & Mortelmans, D. (2021). A preliminary meta-analysis of the Big Five personality traits’ effect on marital separation. *Journal of Divorce & Remarriage,**62*(7), 551–571. 10.1080/10502556.2021.1993018

[CR135] Sutin, A. R., Stephan, Y., & Terracciano, A. (2018). Facets of conscientiousness and objective markers of health status. *Psychology & Health,**33*(9), 1100–1115. 10.1080/08870446.2018.1464165 29718717 10.1080/08870446.2018.1464165 PMC6286646

[CR136] Tavares, L. P. (2016). Who delays childbearing? The associations between time to first birth, personality traits and education. *European Journal of Population,**32*(4), 575–597. 10.1007/s10680-016-9393-1 30976223 10.1007/s10680-016-9393-1 PMC6241015

[CR137] Thomson, E., & Eriksson, H. (2013). Register-based estimates of parents’ coresidence in Sweden, 1969–2007. *Demographic Research,**29*, 1153–1186. 10.4054/DemRes.2013.29.42

[CR138] Todesco, L. (2011). A matter of number, age or marriage? Children and marital dissolution in Italy. *Population Research and Policy Review,**30*(2), 313–332. 10.1007/s11113-010-9190-1

[CR139] Todesco, L. (2013). Family social background and marital instability in Italy. Do parental education and social class matter? *The Social Science Journal,**50*(1), 112–126. 10.1016/j.soscij.2012.09.005

[CR140] Uher, J. (2017). Open peer commentary and authors’ response: Comments. *European Journal of Personality,**31*(5), 529–595. 10.1002/per.2128

[CR141] Usslepp, N., Hübner, N., Stoll, G., Spengler, M., Trautwein, U., & Nagengast, B. (2020). RIASEC interests and the Big Five personality traits matter for life success—But do they already matter for educational track choices? *Journal of Personality,**88*(5), 1007–1024. 10.1111/jopy.12547 32145064 10.1111/jopy.12547

[CR142] van de Kaa, D. J. (1987). Europe’s Second Demographic Transition. *Population Bulletin,**42*(1), 3–55. 12268395

[CR143] Van Gestel, S., & Van Broeckhoven, C. (2003). Genetics of personality: Are we making progress? *Molecular Psychiatry,**8*(10), 840–852. 10.1038/sj.mp.4001367 14515135 10.1038/sj.mp.4001367

[CR144] Vukasović, T., & Bratko, D. (2015). Heritability of personality: A meta-analysis of behavior genetic studies. *Psychological Bulletin,**141*(4), 769–785. 10.1037/bul0000017 25961374 10.1037/bul0000017

[CR145] Wiik, K. A., Bernhardt, E., & Noack, T. (2010). Love or money?: Marriage intentions among young cohabitors in Norway and Sweden. *Acta Sociologica,**53*(3), 269–287. 10.1177/0001699310374488

[CR146] Willitts, M., Benzeval, M., & Stansfeld, S. (2004). Partnership history and mental health over time. *Journal of Epidemiology and Community HealTh,**58*(1), 53–58. 10.1136/jech.58.1.53 14684727 10.1136/jech.58.1.53 PMC1757022

[CR147] Wright, M. R., & Brown, S. L. (2017). Psychological well-being among older adults: The role of partnership status: Psychological well-being among older adults. *Journal of Marriage and Family,**79*(3), 833–849. 10.1111/jomf.12375 28626245 10.1111/jomf.12375 PMC5469370

[CR148] Zare, B., Nasir, R., Mastor, K. A., & Shahrazad, W. S. W. (2013). Personality traits, the risk of divorce and marital satisfaction: An intrapersonal model. *The Social Sciences,**8*(5), 466–472.

